# ANNarchy: a code generation approach to neural simulations on parallel hardware

**DOI:** 10.3389/fninf.2015.00019

**Published:** 2015-07-31

**Authors:** Julien Vitay, Helge Ü. Dinkelbach, Fred H. Hamker

**Affiliations:** ^1^Department of Computer Science, Chemnitz University of TechnologyChemnitz, Germany; ^2^Bernstein Center for Computational Neuroscience, Charité University MedicineBerlin, Germany

**Keywords:** neural simulator, Python, rate-coded networks, spiking networks, parallel computing, code generation

## Abstract

Many modern neural simulators focus on the simulation of networks of spiking neurons on parallel hardware. Another important framework in computational neuroscience, rate-coded neural networks, is mostly difficult or impossible to implement using these simulators. We present here the ANNarchy (Artificial Neural Networks architect) neural simulator, which allows to easily define and simulate rate-coded and spiking networks, as well as combinations of both. The interface in Python has been designed to be close to the PyNN interface, while the definition of neuron and synapse models can be specified using an equation-oriented mathematical description similar to the Brian neural simulator. This information is used to generate C++ code that will efficiently perform the simulation on the chosen parallel hardware (multi-core system or graphical processing unit). Several numerical methods are available to transform ordinary differential equations into an efficient C++code. We compare the parallel performance of the simulator to existing solutions.

## 1. Introduction

The efficiency and flexibility of neural simulators becomes increasingly important as the size and complexity of the models studied in computational neuroscience grows. Most recent efforts focus on spiking neurons, either of the integrate-and-fire or Hodgkin-Huxley type (see Brette et al., 2007, for a review). The most well-known examples include Brian (Goodman and Brette, 2008; Stimberg et al., 2014), NEST (Gewaltig and Diesmann, 2007), NEURON (Hines and Carnevale, 1997), GENESIS (Bower and Beeman, 2007), Nengo (Bekolay et al., 2014), or Auryn (Zenke and Gerstner, 2014). These neural simulators focus on the parallel simulation of neural networks on shared memory systems (multi-core or multi-processor) or distributed systems (clusters) using either OpenMP (open multi-processing) or MPI (message parsing interface). Recent work address the use of general-purpose graphical processing cards (GPU) through the CUDA or OpenCL frameworks (see Brette and Goodman, 2012, for a review). The neural simulators GeNN^1^, NCS (Thibeault et al., 2011), NeMo (Fidjeland et al., 2009), and CARLsim (Carlson et al., 2014) provide in particular support for the simulation of spiking and compartmental models on single or multiple GPU architectures.

A common approach to most of these neural simulators is to provide an extensive library of neuron and synapse models which are optimized in a low-level language for a particular computer architecture. These models are combined to form the required network by using a high-level interface, such as a specific scripting language (as in NEST or NEURON) or an interpreted programming language (e.g., Python). As these interfaces are simulator-specific, the PyNN interface has been designed to provide a common Python interface to multiple neural simulators, allowing a better exchange of models between researchers (Davison et al., 2008). The main drawback of this approach is that a user is limited to the neuron and synapse models provided by the simulator: if one wants to even marginally modify the equations of a model, one has to write a plugin in a low-level language without breaking the performance of the simulator. This can be particularly tedious, especially for CUDA code on GPUs.

A notable exception is the Brian simulator, which allows the user to completely define the neuron and synapse models using a simple mathematical description of the corresponding equations. Brian uses a code generation approach to transform these descriptions into executable code (Goodman, 2010), allowing the user to implement any kind of neuron or synapse model. The first version of Brian executes the code in Python directly (although some code portions can be generated in a lower-level language) using vectorized computations (Brette and Goodman, 2011), making the simulation relatively slow and impossible to run in parallel on shared memory systems. The second version in development (Brian 2, Stimberg et al., 2014) proposes a complete code generation approach where the simulation can be implemented in different languages or parallel frameworks. This approach is promising as it combines flexibility in model design with efficient and parallel simulation performance.

Rate-coded networks, however, do not benefit much from the advances of spiking simulators. Rate-coded neurons do not communicate through discrete spike events but through instantaneous firing rates (real values computed at each step of the simulation). Rate-coded simulators are either restricted to classical neural networks (static neurons learning with the backpropagation algorithm) or optimized for particular structures such as convolutional networks. To our knowledge, no rate-coded simulator provides a flexibility similar to what Brian proposes. The Emergent simulator (Aisa et al., 2008) provides some features—including parallel computing—and is used in a number of models in computational neuroscience (e.g., O'Reilly and Frank, 2006) but is restricted to a set of neuron and synapse models provided by the Leabra library. Topographica (Bednar, 2009) and CNS (Cortical Network Simulator, Mutch et al., 2010) primarily focus on convolutional networks. DANA (Distributed, Asynchronous, Numerical and Adaptive computing framework, Rougier and Fix, 2012) is a generic solver for distributed equations which can flexibly simulate dynamical rate-coded networks, but it does not address parallel computing yet.

Rate-coded networks are nevertheless an important paradigm in computational neuroscience, as they allow to model complex structures and dynamics with a smaller computational footprint than spiking networks. Each unit of a rate-coded network can model the dynamics of several biological neurons, so a rate-coded network typically requires less units to perform a function than a functionally equivalent spiking network. The rate-coded domain also benefits from a wide range of biologically realistic learning rules—such as the Bienenstock-Cooper-Munro (BCM) rule (Bienenstock et al., 1982) or the Oja learning rule (Oja, 1982). Synaptic plasticity in spiking networks, including spike-timing dependency plasticity (STDP), is an active research field and the current implementations can be hard to parameterize. Except in cases where synchronization mechanisms take place or where precise predictions at the single-cell level are required, rate-coded networks can provide a valid approximation of the brain's dynamics at the functional level, see for example models of reinforcement learning in the basal ganglia (O'Reilly and Frank, 2006; Dranias et al., 2008; Schroll et al., 2014), models of visual attention (Zirnsak et al., 2011; Beuth and Hamker, 2015) or models of gain normalization (Carandini and Heeger, 2012).

Another reason why rate-coded networks should not be neglected by neural simulators is that advances in computational neuroscience allow to aim at complete functional models of the brain which could be implemented in simulated agents or robots (e.g., Eliasmith et al., 2012). However, spiking networks may not yet be able to perform all the required functions, especially when in a learning context. Hybrid architectures, combining rate-coded and spiking parts, may prove very useful to achieve this goal. We consider there is a need for a parallel neural simulator which should: (1) be flexible for the definition of neuron and synapse models, (2) allow the definition of rate-coded, spiking and hybrid networks, (3) be computationally efficient on CPU- and GPU-based hardware and (4) be easy to interface with external programs or devices (such as robots).

This article presents the neural simulator ANNarchy (Artificial Neural Networks architect) which allows to simulate rate-coded, spiking as well as hybrid neural networks. It proposes a high-level interface in Python directly inspired from PyNN for the global structure and Brian for the definition of neuron and synapse models. It uses a C++ code generation approach to perform the simulation in order to avoid the costs of an interpreted language such as Python. Furthermore, rate-coded and spiking networks raise different problems for parallelization (Dinkelbach et al., 2012), so code generation ensures the required computations are adapted to the parallel framework. ANNarchy is released under the version 2 of the GNU Public License. Its source code and documentation^2^ are freely available.

## 2. Interface of the simulator

### 2.1. Structure of a network

The interface of ANNarchy focuses on the definition of populations of neurons and their interconnection through projections. Populations are defined as homogeneous sets of identical neurons, while projections gather all synapses formed between the neurons of the pre-synaptic population and the ones of the post-synaptic population. Each projection is associated to a target name (e.g., “exc” for excitatory synapses and “inh” for inhibitory ones). This allows the post-synaptic neurons receiving these synapses to integrate them differently, for example to implement modulatory effects. The target can represent the excitatory/inhibitory nature, the corresponding neurotransmitter (“ampa,” “nmda,” “gaba”) or even the functional role of a synapse (“feedforward,” “feedback”).

**Figure 1:**
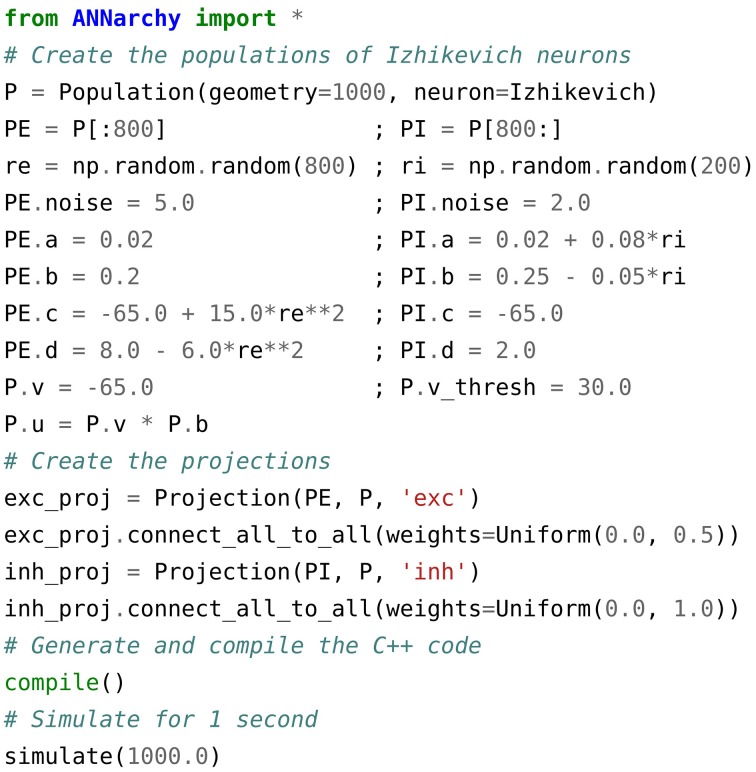
**ANNarchy script reproducing the pulse-coupled spiking network described in Izhikevich (2003)**. A population of 1000 Izhikevich neurons is created and split into subsets of 800 excitatory and 200 inhibitory neurons. The different parameters of the Izhikevich neuron are then initialized through attributes of the two populations. a, b, c, and d are dimensionless parameters, noise is a multiplicative factor on the random variable Normal(0., 1.) drawn each step from the standard normal distribution 𝒩(0,1), v_thresh is the spiking theshold of the neurons and tau is the time constant in milliseconds of the membrane conductances. The network is fully connected, with weight values initialized randomly using uniform distributions whose range depend on the pre-synaptic population. The source code for the network is then generated, compiled and simulated for 1000 ms.

Figure 1 shows a simple example implementing the pulse-coupled spiking network proposed by Izhikevich (2003). It creates a population of 1000 Izhikevich neurons and splits it into two subsets of 800 excitatory and 200 inhibitory neurons each. These neurons are reciprocally connected with each other (all-to-all connection pattern) through excitatory and inhibitory synapses. Such a pulse-coupled network exhibits oscillating pattern at various frequencies, depending on the strength of the connections. The example uses Izhikevich neurons, which are defined by Equation (1):

(1) $$\begin{array}{l} \;I(t)=g_{\text{exc}}(t)-g_{\text{inh}}(t)+n\cdot \chi \\ \;\frac{dv(t)}{dt}=0.04\cdot v{\left(t\right)}^{2}+5\cdot v(t)+140-u(t)+I(t)\\ \;\frac{du(t)}{dt}=a\cdot (b\cdot v(t)-u(t))\\ \text{if }v(t)>v_{\text{thresh}}:\;v(t)=c\text{ and }u(t)\text{ += }d \end{array}$$

with *I*(*t*) being the total input current to a neuron at time *t*, *g*_exc_(*t*) (resp. *g*_inh_(*t*)) the total current current injected by excitatory (resp. inhibitory) synapses, *v*(*t*) the membrane potential and *u*(*t*) a recovery variable. χ is an additive random variable following a standard normal distribution and *n* a multiplicative factor. When the membrane potential *v*(*t*) exceeds a threshold *v*_thresh_, a spike is emitted, the membrane potential is reset and the recovery variable is incremented. *a*, *b*, *c*, and *d* are dimensionless parameters specifying the dynamics of the neuron type.

Populations are defined by three fields: (1) the geometry, which can represent either the total number of neurons (a single integer) or a multi-dimensional structure (tuple) similar to the shape of a Numpy array (van der Walt et al., 2011); (2) the type of neuron used in the population (either a pre-defined neuron model or one defined by the user, see Sections 2.3 and 2.4) and (3) an optional unique name allowing to access the population globally. Defining a multi-dimensional geometry is primarily useful for visualization purposes and when defining distance-dependent connection patterns between two populations, but the internal data is arranged in one-dimensional arrays (see Section 3.1).

Once the populations are created, the value of each parameter and variable can be directly set using population attributes, by providing either a single value (which will be the same for all neurons) or lists/Numpy arrays of the same size/shape as the population. Like many other simulators, but unlike Brian, parameters and variables use implicit physical units: except for time which is expressed in milliseconds, the user must decide if the value of a variable represents volts or millivolts, for example. Brian uses explicit physical units, which allows to ensure consistency between the parameters. The neurons of a population can be accessed either individually or in subsets (similar to the PopulationViews of PyNN), allowing a finer control over the parameter values. Subsets use the slice notation of NumPy.

Projections are defined by four values: (1) the pre-synaptic population, (2) the post-synaptic population, (3) the associated target (e.g., “exc” or “inh”) and (4) optionally the synapse type. Subsets of a population can also be used to create the projection. A connecting method has to be applied on the projection in order to create the synapses using a pre-defined scheme and initialize the corresponding weights and delays. The network is here fully connected, using the connect_all_to_all() method. Several methods are provided by the simulator (all-to-all, one-to-one, distance-dependent, probabilistic…) but the user can also define its own connection patterns in Python, or load connection matrices from a file. Compatibility with the *Connection Set Algebra* proposed by Djurfeldt (2012) is currently under development.

Once the populations and projections are defined and initialized, the corresponding C++code has to be generated and compiled by calling the compile() method. If the network structure has not changed since the last execution of the script, compilation is skipped. The C++ structures storing the parameters and variables of the populations and projections are then initialized with the values previously defined. The network can be then simulated for a certain duration in milliseconds. The values of all population/projection attributes can be read and modified at any point between two calls to simulate(), allowing an easy definition of complex experimental protocols.

This simple script outlines the high-level interface necessary to create a network: in its most simple form, all implementation details (including the neuron/synapse models) are hidden to the user. At this level, there is also no distinction between rate-coded and spiking networks. This distinction only appears when defining or using neuron and synapse models.

### 2.2. Equation-oriented description

Neuron and synapse models are described using an equation-oriented approach, where each equation is expressed by a simple textual description. The goal of the syntax is to provide a high flexibility to the user while being close to natural mathematical descriptions (Stimberg et al., 2014). Our equation-oriented syntax has been designed to be close to the Brian syntax (Goodman and Brette, 2008), although some differences had to be introduced to take into account the semantic difference between rate-coded and spiking neurons.

The syntax chosen for the equations ruling each variable allows to describe most common mathematical operations. Each variable has to be described by an equation, either regular or differential. For the moment, ANNarchy only supports first-order ordinary differential equations (ODE). For regular equations, the left side must hold only the name of the variable which will be updated (e.g., a = b + c). The available operators are assignment (=) and the different augmented assignments (+=, -=, *=, /=). For ODEs, the left term can be more complex (tau*dv/dt + v = E is the same as dv/dt = (E - v)/tau), but only the assignment operator is allowed. The right term can use single operations (+, -, *, /) or power functions (y^d) of other parameters or variables. Different mathematical functions are available (given they exist in the C math library), for example cos, sin, exp, log…

Conditional statements (if/then/else) can be useful for some rate-coded neurons, although they are classically avoided in spiking neurons. They follow a Python-like syntax using the if and else keywords and : as a separator. The rectifier transfer function can for example be implemented like this:


r = if v > 0.0: v else: 0.0


with r being the output of a neuron and v its net activation. The condition can use any parameters or variable of the neuron or synapse. All relational operators are available (<, >, <=, >=, ==, !=…), and they can be combined using the and and or logical operators. Conditioal statements can be nested.

### 2.3. Rate-coded neurons and synapses

#### 2.3.1. Rate-coded neurons

**Figure 2:**
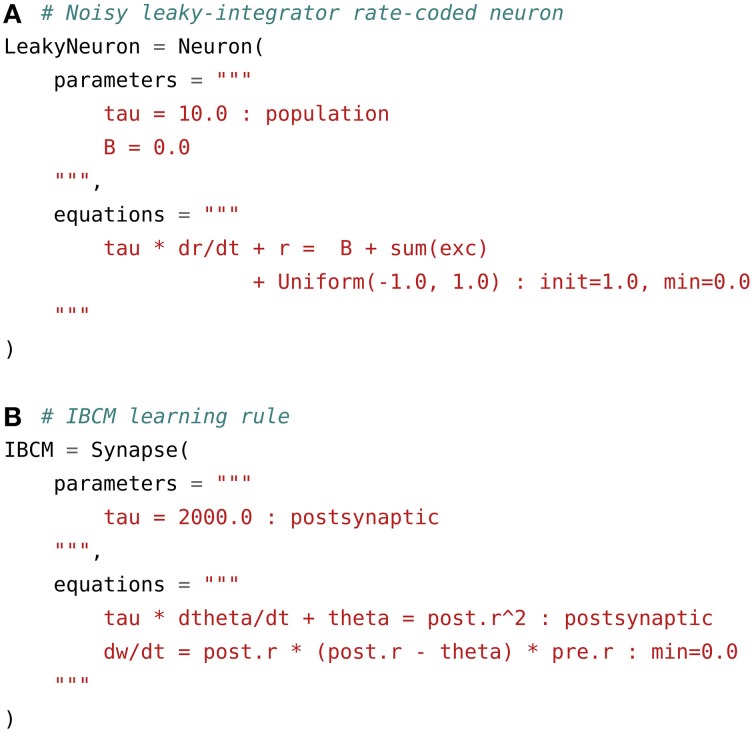
**Examples of rate-coded neuron and synapse definitions**. **(A)** Noisy leaky-integrator rate-coded neuron. It defines a global parameter tau for the time constant and a local one B for the baseline firing rate. The evolution of the firing rate r over time is rules by an ODE integrating the weighted sum of excitatory inputs sum(exc) and the baseline. The random variable is defined by the Uniform(–1.0, 1.0) term, so that a value is taken from the uniform range [−1, 1] at each time step and for each neuron. The initial value at *t* = 0 of r is set to 1.0 through the init flag and the minimal value of r is set to zero. **(B)** Rate-coded synapse implementing the IBCM learning rule. It defines a global parameter tau, which is used to compute the sliding temporal mean of the square of the post-synaptic firing rate in the variable theta. This variable has the flag postsynaptic, as it needs to be computed only once per post-synaptic neuron. The connection weights w are then updated according to the IBCM rule and limited to positive values through the min=0.0 flag.

The definition of a rate-coded neuron model is done by instantiating a Neuron object, with arguments specifying the parameters and variables of the neuron. Let us consider a simple noisy leaky-integrator rate-coded neuron:

(2) $$\tau \cdot \frac{dr(t)}{dt}+r(t)=\sum_{i=1}^{N}w_{i}\cdot r_{i}(t)+B(t)+𝒰(-1,1)$$

where *r*(*t*) is the instantaneous firing rate of the neuron at time *t*, τ its time constant, *B*(*t*) its baseline firing rate (which can change over time), 𝒰(−1, 1) a random variable taken at each time *t* in the uniform range [−1, 1] in order to add noise and $\sum_{i=1}^{N}w_{i}\cdot r_{i}$ represents the weighted sum of excitatory inputs to a particular neuron. Figure 2A shows a possible implementation of such a neuron in ANNarchy.

The first argument parameters is a string or multi-line string defining two parameters: tau, the time constant of the neuron, initialized to 10 ms, and B, the baseline firing rate, initialized to 0. Parameter definitions can be placed on different lines or separated by semi-colons. Once a population is created, these parameters are accessible and modifiable through population attributes. Various flags can be set after the : symbol. In this example, the flag population tells the code generator that the value of tau will be shared by all neurons of a population, so it only needs to store one value. It is also possible to specify the type of the parameter: parameters (and variables) are by default represented by double precision floating-point values. The int and bool flags change the type of the attribute to integer or boolean, if needed.

The second argument equations defines the variables of the neuron, whose value will evolve with time during the simulation. The number of variables defined in the model is unlimited, but at least one of them should be named r, as this is the default variable used by post-synaptic neurons to compute their weighted sum of inputs. The code corresponding to Equation (2) is straightforward. The temporal derivative of *r*(*t*) is symbolized by the term dr/dt. The random variable 𝒰(−1, 1) is generated by the term Uniform(-1.0, 1.0), where –1.0 and 1.0 and the bounds of the uniform range. Different distributions can be used in an equation, including the normal, log-normal, exponential and gamma distributions. The weighted sum of excitatory inputs is represented by sum(exc), which sums over all projections possibly reaching a particular neuron the product between the connection weight w and the firing rate of the pre-synaptic neuron r. The term exc corresponds to the target name defined when creating the projections. By default, this ODE will be solved using the explicit (forward) Euler method, but other methods are available, see Section 3.4. The flag init defines the initial value of the variable for all neurons and min defines a lower bound for the variable (if r is negative after an update, it will be set to 0), as the firing rate r is usually ensured positive in rate-coded networks. The max flag is also available.

#### 2.3.2. Rate-coded synapses

When the pre-synaptic population of a projection is rate-coded, the synapses of the projection are assumed to be also rate-coded. A synapse is represented by a fixed connection weight (or synaptic efficiency) named w and a delay in synaptic transmission d (in milliseconds). Each synapse will participate in the weighted sum of inputs of the post-synaptic neuron with *w*(*t*) * *r*(*t* − *d*), where *r*(*t* − *d*) is the firing rate of the pre-synaptic neuron at time *t* − *d*. Synaptic delays in a network must be a multiple of the fixed integration step dt (see Section 3.4), but each synapse of a projection can define a different delay. The minimal delay is dt, as neurons can only access the value of variables computed at the previous time step (synchronous computation). Note that the Brian simulator can simulate rate-coded synapses, but only without delay.

In a learning context, connection weights evolve with time according to a variety of learning rules (Dayan and Abbott, 2001). Synapse models can be created to override the default behavior and implement synaptic plasticity or non-linear transmission. Figure 2B shows a possible implementation of the IBCM learning rule (Intrator and Cooper form of the BCM rule) (Intrator and Cooper, 1992). It is a Hebb-like product of the pre-synaptic firing rate and a quadratic function of the post-synaptic firing rate. The quadratic function uses a dynamical threshold θ(*t*) which is defined as the expectation of the square of the post-synaptic firing rate:

(3) $$\begin{array}{l} \;\theta (t)=E(y^{2}(t))\\ \frac{dw(t)}{dt}=y(t)\cdot (y(t)-\theta (t))\cdot x(t) \end{array}$$

where *x*(*t*) is the pre-synaptic firing rate, *y*(*t*) the post-synaptic one, *w*(*t*) the connection weight and θ(*t*) is defined as the moving average of *y*^2^(*t*) through the *E*() expectation operator. In the code displayed on Figure 2B, the moving average is calculated using a first-oder ODE integrating the square of the post-synaptic firing rate, with a time constant tau of 2 s by default. Pre- and post-synaptic neural variables (usually the firing rate r, but any other variable can be used) can be accessed by prefixing the variable name by pre. and post., respectively.

The update rule for the weight w is simply derived from Equation (3) using these conventions. theta is a post-synaptic variable, as it only depends on the post-synaptic neural activity. It would therefore be a waste of resources to compute it for each synapse: once per post-synaptic neuron is enough. The equation for theta (as well as the corresponding parameter tau) is associated with the flag postsynaptic, which has a similar meaning as population for a neuron: the global variable will be updated only once per post-synaptic neuron. The variable w is local to a synapse, so the flag should not be set. Instead, min=0.0 is used to ensure that the weight will not become negative over time.

In a rate-coded neuron model, the term sum(exc) represents by default the weighted sum of excitatory inputs to this neuron. It is possible to change this behavior in the synapse definition by adding a psp argument to the synapse definition, whose default value is "w * pre.r". Non-linear synapses, where for example *w*_i_ · log(*r*_*i*_) should be summed over all synapses instead of *w*_*i*_ · *r*_*i*_, can be implemented by setting psp = "w * log(pre.r)". The summation operation can also be changed, by defining the operator argument, whose default value is "sum". If "max", "min" or "mean" is used, the maximal (resp. minimal or mean) value of psp is calculated over all synapses associated to the target exc will be returned by sum(exc). This is particularly useful for pooling operations, which are used for example in hierarchical visual processing (Riesenhuber and Poggio, 1999; Hamker, 2004).

### 2.4. Spiking neurons and synapses

#### 2.4.1. Spiking neurons

**Figure 3:**
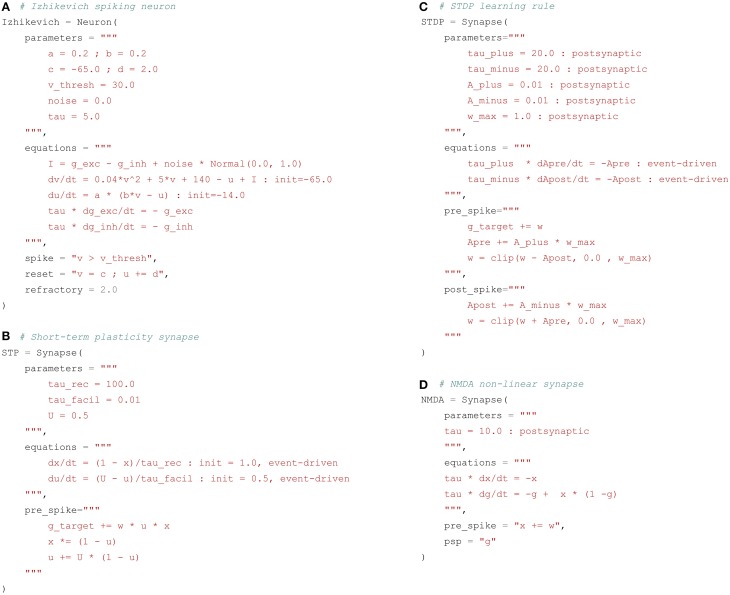
**Examples of spiking neuron and synapse definitions**. **(A)** Izhikevich neuron. The parameters and equations fields follow the same principles as for rate-coded neurons. The variable I gathers the inputs to the neuron, namely the sum of the excitatory g_exc and inhibitory g_inh input currents and a constant current i_offset. The membrane potential v and the recovery variable u are updated according to the desired dynamics, with initial values specified with the init keyword. The spike field defines the condition for emitting a spike, here when the membrane potential v exceeds the threshold v_thresh. The reset field specifies the modifications happening after a spike is emitted. Here the membrane potential is clamped to the value c and the recovery variable u is incremented by d. The refractory period is determined by the refractory field, here 2 ms. **(B)** Short-term plasticity (STP) synapse. For this synapse, the increment of the post-synaptic conductance g_target when a pre-synaptic spike arrives depends not only on the synaptic efficiency w, but also on the value of variables internal to the synapse x and u. These are updated through two mechanisms: the equations field specifies their exponentially-decreasing dynamics, while the pre_spike defines their increments when a pre-synaptic spike arrives at the synapse. However, the integration of the corresponding ODEs is event-driven through the use of the event-driven flag: when a pre- or post-synaptic spikes occurs, the new value of these variables is directly computed using the analytical solution of the ODE. This can speed up the simulation if the number of spiking events is low. **(C)** Spike-timing dependent plasticity (STDP) synapse. For this synapse, the post-synaptic conductance is increased by w after a pre-synaptic spike is received, but the synaptic efficiency is adapted depending on two internal variables Apre and Apost. The pre_spike field states what should happen when a pre-synaptic spike arrives at the synapse, while the post_spike field describes the changes occuring when the post-synaptic neuron fires. The variables Apre and Apost are integrated in an event-driven manner. The clip() function is used to maintain w in the range [0, w_max]. **(D)** NMDA non-linear synapse. This synapse does not transmit information to the post-synaptic neuron in an event-driven manner. Rather, the synaptic variable g is summed at each time step by the post-synaptic neuron, as for rate-coded networks. This is specified by the psp field. When a pre-synaptic spike occurs, the variable x is increased by w, which in turn will modify the evolution of g through the coupled equations described in the equations field. These equations cannot be solved with the event-driven method, as their values should be available at each time step.

Integrate-and-fire neurons (IF) describe the temporal evolution of the membrane potential *v*(*t*) through a system of first-order ODEs. When the membrane potential exceeds a given threshold, a spike is emitted and the value of the different neural variables is clamped to a reset value for a certain duration called the refractory period. The condition for spike emission as well as the reset and refractory behaviors have to be explicitly defined in addition to the internal dynamics. More complex spiking neurons such as the Hodgkin-Huxley neuron model have their own dynamics for the reset and refractory mechanisms. Figure 3A shows a possible implementation of the Izhikevich neuron described by Equation (1).

As for rate-coded neurons, the argument parameters describes the different parameters of the neuron model: a, b, c and d are dimension-less parameters, v_thresh is the spiking threshold, noise is a multiplying factor on the noise random variable and tau is the time constant in milliseconds of the conductances. The argument equations describes the evolution of the three variables I, v and u of Equation (1). Normal(0., 1.) is a random variable taken fromthe standard normal distribution. g_exc and g_inh represent the total excitatory and inhibitory currents or conductances generated by incoming pre-synaptic spikes. They are the equivalent for spiking neurons of sum(exc) and sum(inh) for rate-coded neurons. The syntax g_target is different from the rate-coded case because they have a different behavior: while sum(target) is computed at every time step of the simulation by summing pre-synaptic activity, g_target is event-driven. Every time a pre-synaptic spike arrives to a neuron, the corresponding conductance is increased from a value corresponding to the weight (or efficiency) w of the synapse. If no spike arrives, the conductance evolves with its own dynamics, independently from inputs.

The default behavior for conductances is governed by instantaneous synapses: once all the incoming spikes have been summed, the total conductance is reset to 0 for the next time step. More realistic models use exponentially decreasing or alpha (double exponential) functions to model the dynamics of the conductance. The example of Figure 3A uses exponentially decreasing synapses, by specifying a linear first-order ODE for the conductances g_exc and g_inh. If no spike arrives for a certain duration, the conductances will progressively decay back to 0, with a time constant defined by the parameter tau.

Two other arguments of the Neuron object have to be defined: spike defines the spiking condition, i.e., the condition that must be satisfied in order to emit a spike (typically when the membrane potential exceeds a given threshold); reset describes what should happen after a spike is emitted. The spiking condition has to be a boolean expression; it can depend on any parameter or variable, possibly combined through the logical operators and and or. The reset statement forces some neural variables to take predefined values after a spike is emitted: here the membrane potential is clamped to a reset value c and the recovery variable is incremented by d.

Spiking neurons can also define a refractory period, during which the ODEs are not evaluated (i.e., the membrane potential stays at its reset value), except for the conductances g_exc and g_inh. This corresponds to the hyper-polarized state of a neuron after spike emission, where no spike can be further emitted. The duration of this refractory period is set through the refractory argument, which takes here a constant value of 2 ms, but the name of a parameter or variable can be given, allowing for dynamical refractory period: for example, the refractory period can be progressively increased if the firing rate becomes too high.

As shown in Stimberg et al. (2014), the five arguments parameters, equations, spike, reset, and refractory are sufficient to describe the dynamics of most point-spiking neurons, including IF and Hodgkin-Huxley models, and are directly related to the Brian syntax (although parameters is implicit in Brian). They are not well suited to describe multi-compartment models, which are the main focus of simulators such as NEURON or GENESIS. However, Brian 2 introduces support for this kind of models.

#### 2.4.2. Event-driven synaptic transmission

Synaptic behavior in spiking networks is also different from rate-coded networks, and requires additional description. The basic type of synapses is the linear synapse, where synaptic transmission is event-driven: when the pre-synaptic neuron emits a spike, it increases the corresponding post-synaptic conductance by a given value (generally the synaptic efficiency w). If no spike occurs, the synapse does not need to transmit any information: the dynamics of conductances are already defined at the post-synaptic neuron level. As in Brian, a spiking synapse can therefore define two additional arguments: pre_spike which specifies what should happen when a pre-synaptic spike arrives at the synapse (potentially after a given delay) and post_spike when the post-synaptic neuron emits a spike. The default linear synapse only defines pre_spike with the value g_target += w. g_target is a generic name for the conductance associated to the synapse. Depending on the target of the projection, g_target will be replaced by g_exc or g_inh, for example. The underlying idea is that the same synapse type can be used in different projections, regardless of their target.

Some event-driven synapse models modify the post-synaptic conductance with a value depending on specific synaptic variables. This is for example the case in short-term plasticity (STP) synapses (Markram et al., 1998), where the increment of the post-synaptic conductance depends on the history of the synapse. Frequent stimulation of a facilitating synapse leads to an increased influence on the post-synaptic neuron, while depressing synapses show the opposite effect. A possible model of STP synapses uses two internal variables *u*(*t*) and *x*(*t*), which evolve continuously according to linear ODEs:

(4) $$\begin{array}{l} \;\tau_{\text{rec}}\cdot \frac{dx(t)}{dt}=1-x(t)\\ \tau_{\text{facil}}\cdot \frac{du(t)}{dt}=U-u(t) \end{array}$$

When a pre-synaptic spike arrives at the synapse, the post-synaptic conductance should be incremented with *w*(*t*) · u(*t*) · x(*t*), while the synaptic variables should be modified according to:

(5) $$\begin{array}{l} x(t)\leftarrow x(t)\cdot (1-u(t))\\ u(t)\leftarrow u(t)+U\cdot (1-u(t)) \end{array}$$

Figure 3B shows an implementation of a synapse with short-term plasticity. The parameters are tau_rec, tau_facil, and U, which define the dynamics of the synapse and whether it is facilitating or depressing. The two variables u and x directly relate to Equation (4). The pre_spike argument defines what should be modified when the pre-synaptic spike occurs: g_target should be incremented with w*u*x instead of w by default, and u and x are modified according to Equation (5).

The equations for u and x use the flag event-driven. As explained later in Section 3.4, this defines the numerical method used to integrate the ODE. Here both variables are defined by first-order linear ODEs, so their current value can be directly calculated whenever a pre- or post-synaptic spike occurs, based on the time elapsed since the last event (exponentially decreasing function of time). This can spare a lot of computations if the number of spikes in the network is not very high.

An event-driven synapse does not need to rely only on spike times for its dynamics. As for rate-coded synapses, it can access pre- and post-synaptic variables during updates: the pre- (resp. post-) synaptic membrane potential is accessed with pre.v (resp. post.v). Pre-synaptic variables are delayed if necessary. However, only the post-synaptic conductance g_target can be modified by a synapse, contrary to Brian 2.

#### 2.4.3. Synaptic plasticity

Synaptic plasticity can also be described using event-driven mechanisms: the weight w of a synapse usually only needs to be updated when a pre- or post-synaptic spike occurs. Most biologically-realistic synaptic plasticity mechanisms in spiking networks indeed derive from the *spike timing dependent plasticity (STDP) rule* (Gerstner et al., 1996; Markram et al., 1997). Although many different implementations exist, there is an online version of STDP which is event-driven (Song et al., 2000). With this rule, each synapse integrates two variables *A*_pre_(*t*) and *A*_post_(*t*) which represent traces of the pre- and post-synaptic spikes, respectively. Between two spikes, they follow linear first-order ODEs:

(6) $$\begin{array}{l} \;\tau_{+}\cdot \frac{dA_{\text{pre}}(t)}{dt}=-A_{\text{pre}}(t)\\ \tau_{-}\cdot \frac{dA_{\text{post}}(t)}{dt}=-A_{\text{post}}(t) \end{array}$$

When a pre-synaptic spike occurs, the pre-synaptic trace *A*_pre_(*t*) is incremented by a fixed value, and at the same time the post-synaptic trace *A*_post_(*t*) is substracted from the synaptic efficiency *w*(*t*), allowing long-term depression (LTD):

(7) $$\begin{array}{l} A_{\text{pre}}(t)\leftarrow A_{\text{pre}}(t)+A_{+}\cdot w_{\text{max}}\\ \;w(t)\leftarrow w(t)-A_{\text{post}}(t) \end{array}$$

with *w*_max_ being the maximal value allowed for the weight. When a post-synaptic spike occurs, the post-synaptic trace is incremented, and the synaptic efficiency *w*(*t*) is increased from the pre-synaptic trace, allowing long-term potentiation (LTP):

(8) $$\begin{array}{l} A_{\text{post}}(t)\leftarrow A_{\text{post}}(t)+A_{-}\cdot w_{\text{max}}\\ \;w(t)\leftarrow w(t)+A_{\text{pre}}(t) \end{array}$$

Figure 3C shows a possible implementation of this STDP plasticity rule. The equations for Apre and Apost can be integrated with an event-driven method, as their value is only required when a pre- or post-synaptic spike occurs. Synaptic transmission is linear, so pre_spike defines g_target += w. The increments in pre_spike and post_spike follow Equations (7) and (8), while the weight w is clipped between 0 and *w*_max_ by using the clip function. An alternative implementation could have used the min and max flags instead of the clip function, as w is a variable of the synapse.

#### 2.4.4. Continuous synaptic transmission

In some cases, synaptic transmission cannot be described in an event-driven framework. Synapses using the NMDA neurotransmitter are for example often modeled as non-linear synapses (Wang, 2002). These synapses require the post-synaptic conductance to be a sum of synapse-specific variables, as for rate-coded neurons, and not simply incremented when a pre-synaptic spike occurs. This is similar to the summed flag of Brian 2. NMDA synapses can be represented by two variables *x*(*t*) and *g*(*t*) following first-order ODEs:

(9) $$\begin{array}{l} \tau \cdot \frac{dx(t)}{dt}=-x(t)\\ \tau \cdot \frac{dg(t)}{dt}=-g(t)+x(t)\cdot (1-g(t)) \end{array}$$

When a pre-synaptic spike occurs, *x*(*t*) is incremented by the weight *w*(*t*). However, it does not directly influence the post-synaptic neuron, as the output of a synapse is the signal *g*(*t*). The post-synaptic conductance is defined at each time *t* as the sum over all synapses of the same type of their variable *g*(*t*):

(10) $$g_{\text{exc}}(t)=\sum_{i=1}^{N_{\text{exc}}}g_{i}(t)$$

Figure 3D shows a possible implementation of such a non-linear NMDA synapse. The main difference with the previous models is that it defines a psp argument which means that the post-synaptic conductance should be summed over this value (g in this case) at every time step. It is therefore not possible to use the event-driven scheme for such non-linear synapses. The psp argument can access any synaptic variable, as well as any pre- or post-synaptic variable. For example, it can be used for gap junctions (also called electrical synapses) which do not exchange spikes but directly a function of the pre- and post-synaptic membrane potentials.

### 2.5. Additional features

#### 2.5.1. Standard neurons and synapses

Although the definition of neuron and synapse types is rather simple, the library provides a set of predefined models which can be used directly when creating populations and projections. Spiking neuron models are conveniently standardized, especially since the introduction of the PyNN interface (Davison et al., 2008). Using the PyNN nomenclature for the model names and parameters, ANNarchy provides the main neuron models common to most neural simulators: simple integrate-and-fire neuron, using either exponentially-decaying or alpha-shaped conductances or currents (IF_curr_exp, IF_cond_exp, IF_curr_alpha, IF_cond_ alpha), adaptive integrate-and-fire neurons (Izhikevich, EIF_cond_alpha_isfa_ista, EIF_cond_exp_isfa_ista), or Hodgkin-Huxley neurons (HH_cond_exp). Synapse models include short-term plasticity (STP) and spike-timing dependent plasticity (STDP). Each model is associated with a docstring describing completely the parameters and equations, allowing to easily create a new derivative model. Rate-coded neuron models are less standardized than spiking ones. The library only provides a generic leaky-integrator neuron similar to Equation (2). Rate-coded synapses include the Hebbian learning rule (Hebb), the Oja learning rule (Oja) and the IBCM learning rule described by Equation (3) (IBCM). The available rate-coded models will be extended in future versions.

#### 2.5.2. Specific populations

Specific populations are available to provide functions which are difficult or unnecessarily complicated to implement with single neuron models. The PoissonPopulation class allows to directly create a population of spiking neurons whose spikes are generated from a Poisson distribution. The rate underlying the distribution can be a single value or one value per neuron (homogeneous Poisson process, as the rate for each neuron is constant), or a string expression defining the evolution of rate over time (e.g., '1 + sin(2*pi*t)', heterogenous Poisson process). The SpikeArray class allows to create a population and to specify for each neuron the exact times at which they will emit a spike. These spiking times can be modified between two simulations using attributes.

The ImagePopulation class allows to represent images through the firing rates of a rate-coded population with the same geometry as the image (two-dimensional for grayscale, three for colored images, the last dimension representing the R, G, and B components). Firing rates are normalized between 0 and 1. It relies on the Python Imaging Library (PIL), which allows the use of many file formats, including JPEG. Similarly, the VideoPopulation class allows to grab image streams from webcams and use them as firing rates of a population. It relies on the OpenCV 2.x C++ library to access the desired hardware. Grabbing images has to be explicitly called by the user between two simulations.

#### 2.5.3. Hybrid networks

Apart from the neuron and synapse definitions, there is no difference in the interface between rate-coded and spiking networks: populations and projections behave the same regardless of the framework. It then becomes possible to create hybrid networks, composed of rate-coded and spiking populations interacting with each other. Interaction between the two types of neurons is achieved by introducing specific populations and projections to perform the conversion.

Converting a rate-coded population into a spiking one is straightforward: the output r of the rate-coded population is interpreted as an instantaneous firing rate in Hz and used to generate spikes according to a Poisson distribution. The abovementioned PoissonPopulation object accepts a target argument, stating that the rate of each Poisson neuron is determined by its weighted sum of inputs:


pop1 = Population(1, Neuron(equations="r = 1
        + sin(2*pi*t)"))
pop2 = PoissonPopulation(100, target=’exc’)
proj = Projection(pop1, pop2, ’exc’)
proj.connect_all_to_all(1.0)


The connectivity matrix can have any form, but in the most simple case one single rate-coded neuron should determine the firing rate of a group of spiking neurons (one-to-many pattern). The weight of the connection determines the scaling: a weight of 1.0 means that a pre-synaptic rate of 1.0 will generate Poisson spike trains at 1 Hz. With a weight of 100.0, the train would be at 100 Hz. Other distributions than Poisson will be added in future versions.

Converting a spiking population into a rate-coded one is a much more difficult problem. Estimating neural firing rates from single spike trains instead of averaging over multiple trials is an open issue in neuroscience (Cunningham et al., 2009). The main methods include peri-stimulus time histograms (PSTH, Gerstein and Kiang, 1960), smoothing kernels (Nawrot et al., 1999), Kalman filters (Wu et al., 2004), or Bayesian estimation (Shimokawa and Shinomoto, 2009). All these methods are biased and can only infer firing frequencies in a particular bandwidth. Here, the problem is even more difficult as it has to be performed online during the simulation: in the interval between two spikes of the same neuron, it is not possible to predict the real instantaneous firing rate of the neuron, as future incoming spikes are still unknown.

ANNarchy provides a simple method to infer online the firing rate of a spiking population, using the assumption that a rate-coded neuron usually represents a large group of spiking neurons. The two populations are connected with a specific projection object DecodingProjection and a many-to-one pattern. For example, a single rate-coded neuron could decode the firing rate of a population of 1000 Poisson neurons:


pop1 = PoissonPopulation(1000, rates=100.0)
pop2 = Population(1, Neuron(equations="r=sum(exc)"))
proj = DecodingProjection(pop1, pop2, ’exc’,
       window=10.0)
proj.connect_all_to_all(1.0)


The input sum(target) of a post-synaptic neuron at time *t* is a weighted sum of all spikes received during a sliding window of duration *T* (defined by the argument window), normalized by the total number of synapses to this neuron:

(11) $$\text{sum(target)}(t)=\frac{\text{Weighted sum of spikes received in }[t-T,t]}{T*\text{Number of incoming synapses}}$$

It approximates the mean firing rate in the pre-synaptic population during the last *T* milliseconds. By default, *T* is equal to the simulation step dt, but the decoded rate may be fluctuating if the number of pre-synaptic neurons is too small. One should either increase *T* or apply a low-pass filter to sum(target) in the post-synaptic neuron. The weights of the projection can be used to scale the output firing rate: by default, an input firing rate at 1 Hz leads to sum(target)=1.0.

**Figure 4:**
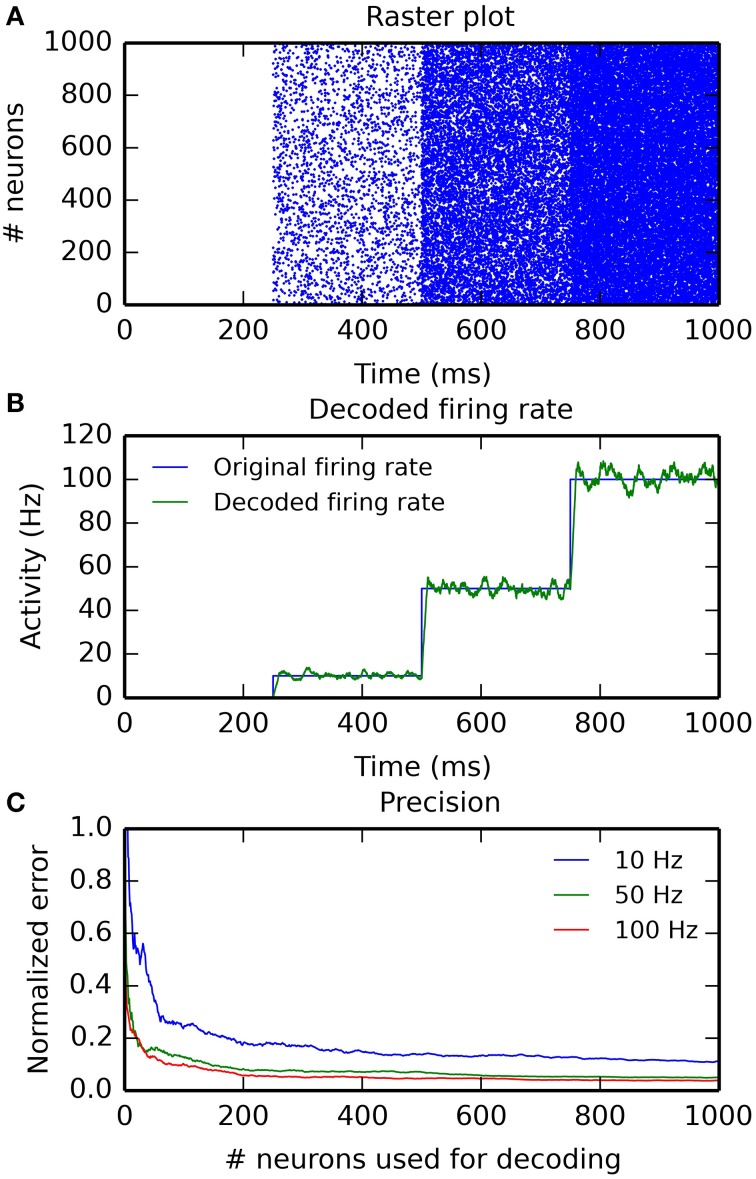
**Example of an hybrid network encoding a rate-coded population into a spiking population (PoissonPopulation) and decoded back to the rate-coded domain (DecodingProjection)**. The script for this plot is provided in the Supplementary Material. **(A)** Raster plot of the spiking population reacting to step-wise inputs for 1 s. Each step lasts 250 ms (0, 10, 50, and 100 Hz). **(B)** Firing rate of a single rate-coded neuron decoding the corresponding spiking neuron. The blue line shows the firing rate in the input population and the green line shows the decoded firing rate. It follows the original firing rate with some noise due to the stochastic nature of the spike trains and some delay due to the integration window. **(C)** Relative decoding error ($\epsilon =\frac{1}{250}\int_{t=0}^{250}\frac{\left|r(t)-F\right|}{F}dt$) depending on the number of spiking neurons used for decoding, for different input firing rates (10, 50, and 100 Hz). For small number of neurons, the decoding error is high as individual spike trains are stochastic. When the number of neurons is increased (over 200), the decoding error is reduced. Decoding is relatively more precise at high frequencies than at low ones.

Figure 4 illustrates the use of hybrid networks. A single rate-coded neuron is used to activate a population of 1000 Poisson neuron with a firing rate increasing every 250 ms (0, 10, 50, and 100 Hz). Figure 4A shows a raster plot of the spikes emitted by the Poisson population. Figure 4B shows the original (blue) and decoded (green) firing rate, for a single rate-coded neuron connected to all 1000 Poisson neurons. The projection uses a sliding window of 10 ms to smoothen the rate. The decoded firing rate follows the original one, but with a small variance due to the stochastic nature of the Poisson spike trains, and with a small temporal lag corresponding to the sliding window: when the firing rate suddenly increases, it takes approximately *T* milliseconds to completely reflect the change.

Figure 4C shows the effect of the number of connected neurons on the precision of the decoding. For the three stimulations at 10, 50, and 100 Hz, we measure the mean of the normalized error between the decoded firing rate *r*(*t*) and its target value *F* ∈ [10, 50, 100]: $\epsilon =\frac{1}{250}\int_{t=0}^{250}\frac{\left|r(t)-F\right|}{F}dt$ for post-synaptic neurons receiving 1–1000 inputs from the Poisson population. Unsurprisingly, the more inputs are used for decoding, the better is the precision. The sliding window method is also more precise at high frequencies, as more spikes can be used to estimate the firing rate. The remaining error for a high number of neurons is mostly due to the temporal lag of the integration. The script allowing to reproduce Figure 4 is given in the Supplementary Material.

#### 2.5.4. Weight sharing and convolution operations

Regular projections instantiate a set of connection weights per post-synaptic neuron. This can be a waste of resources when the weights are identical for each neuron, the only difference being the coordinates of the corresponding neurons in the pre-synaptic population, as it is the case in convolutional networks (Lecun et al., 1998) or image filtering. Such convolution operations can be implemented by creating a SharedProjection instead of a Projection and calling the “convolve()” connector method:


proj = SharedProjection(pre=pop1, post=pop2,
       target=’exc’)
proj.convolve(weights=kernel)


The generated code depends on the respective geometry of the pre- and post-synaptic populations, as well as on the weights kernel. If they all have the same number of dimensions (for example two-dimensional), a regular convolution will be performed:

(12) $${\text{sum}}_{\text{exc}}(x,y)=\sum_{i=-d_{i}}^{d_{i}}\sum_{j=-d_{j}}^{d_{j}}W(i,j)\cdot \text{pre.r}(x-i,y-j)$$

with (*d*_i_, *d*_j_) representing the extent of the weights kernel *W*. If the pre- and post-populations do not have the same number of neurons in each dimension (for example 200 * 200 and 100 * 100, corresponding to a sub-sampling ratio of 2), the mapping between the coordinates of the post-synaptic neurons and the center of the corresponding pre-synaptic region is automatically computed, but this can be overwritten.

The convolution operation can also be performed in parallel over a specific dimension of the pre-synaptic population. For example, if the last dimension of the population represents the RGB color channels of an image, the first two being the width and height, a two-dimensional filter can be applied on each color channel separately. The post-synaptic population has then three dimensions too. It is also possible to apply a bank of filters on the pre-synaptic population (e.g., edge detection with different orientations), leading to a post-synaptic population with one additional dimension (feature map).

Pooling (e.g., max-pooling) can also be implemented using a shared projection. The operation must be specified when creating the projection, before calling the pooling connector method:


proj = SharedProjection(pre=pop1, post=pop2,
       target=’exc’, operation=’max’)
proj.pooling()


Each post-synaptic neuron will be associated to a region of the pre-synaptic population and will extract the maximal firing rate in this region, without defining any weight. For example, if the two populations are 200 * 200 and 100 * 100, each post-synaptic neuron covers a 2 * 2 area. The extent of the region is automatically computed based on the respective geometries, but this can be overwritten. The operation can be changed to the minimal or mean firing rate in the region ('min' and 'mean'). Weight sharing is for the moment only possible for rate-coded networks and learning is disabled. This will be improved in future versions.

#### 2.5.5. Recording of variables

All neural and synaptic variables (defined in the equations argument of a neuron or synapse) can be recorded during a simulation. Populations (or subsets of a population) and projections can be associated to a Monitor object together with a list of variable names. A frequency of recording can also be defined, e.g., once every 10 ms. In the following calls to simulate(), the value of these variables for all neurons/synapses will be internally appended to a vector until get() is called, which returns a matrix containing the recorded values and empties the recording vectors. Recording can be stopped, paused and resumed using methods of Monitor.

The advantage of this recording method is that the user is not bound to a specific file format: the returned values are a dictionary of Numpy arrays (one per variable) which can be directly manipulated or saved into a file. The drawback is that the available RAM can quickly be filled, especially when recording synaptic variables such as weights. It is the user's responsibility to record only the necessary periods of the simulation (using pause/resume) and to save intermediary results regularly.

#### 2.5.6. Conditional simulations

By default, simulate() runs the simulation for a fixed duration. In some cases it may be useful to simulate until a criterion is reached, for example when the maximal firing rate in a population crosses a threshold, or a neuron has emitted a certain number of spikes. This can be used to run conditional simulations, e.g., the network has made a decision and we need to perform the corresponding action. Each population accepts a stop_condition argument, which states the condition that must be true to stop the simulation. In the following example, the simulation would be stopped when one or more neurons of the population have a firing rate r higher than 1:


pop1 = Population( … , stop_condition = "r > 1.0")


The stop condition can use any neural parameter or variable, and can combine several boolean predicates using the and, or, and not operators. If the simulation should be stopped when the condition is true for all neurons, not just any of them, the : all flag can be appended to the condition. The simulation can then be run with the simulate_until() method, which accepts a maximal duration for the simulation (if the criteria is never met) and a (list of) population(s) whose criteria should be checked.

#### 2.5.7. Structural plasticity

The number of synapses in a network is determined at the time when projections are created and is usually constant during the simulation. Some networks require to dynamically add or remove synapses between neurons during the simulation, a mechanism called *structural plasticity* (Butz et al., 2009). Projections define create_synapse() and prune_synapse() methods which allow to dynamically create or delete synapses between any pair of neurons. These functions are called from Python, so the user has to regularly stop the simulation and check if the conditions for creating or deleting a synapse are met, depending on some neural or synaptic variable or randomly. If the structural plasticity mechanism is applied frequently, it will slow down the simulation because of the constant switches between Python and C++.

Alternatively, simple rules for the creation or deletion of a synapse can be passed to the definition of the synapse model. The pruning argument takes a simple boolean expression which, when true, will lead to the online deletion of the synapse. Oppositely, the creating argument defines a binary condition which leads to the creation of a synapse if it does not exist yet. Creation or deletion can be made probabilistic by passing the flag proba after the rule. The weight and delay of created synapses can also be specified.

In the following example, each synapse updates an age variable which is incremented at each simulation step, but is reset to 0 when both pre- and post-synaptic neurons are simultaneously active. When the age of a synapse exceeds a given threshold, the synapse is pruned with a probability of 0.5. Similarly, a synapse can be created when two unconnected neurons are strongly active at the same time.


StructuralPlasticity = Synapse(
      parameters = "max_age = 1000.0 : postsynaptic",
      equations = "age = if pre.r * post.r > 0.9:
       0.0 else: age + dt",
      pruning = "age > max_age : proba=0.5",
      creating = "pre.r * post.r > 0.9 : proba=0.5,
       w=0.5"
)


Creation and pruning of synapses have to be explicitly started with start_creating() and start_pruning() methods, which also accept a period argument defining how often the structural plasticity conditions will be checked (by default at every time step, which is computationally inefficient and probably unnecessary in most cases). Structural plasticity is available for spiking networks, but creating and pruning can not be linked to events such as the emission of a spike: it must rely on continuous variables.

#### 2.5.8. Reporting

As noted by Stimberg et al. (2014), the equation-based representation of neural networks allows the automatic documentation of models. Parameters are known, equations can be parsed to 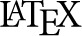 mathematical code, and the structure of the network is simply defined in terms of populations and projections. User-defined neuron or synapse models can be documented by adding a name and a detailed text description of its behavior. Calling the report() method will generate a complete 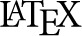 file, organized in tables as suggested by Nordlie et al. (2009). It contains a summary of the network, a list of all the populations (including their size and the neuron model), a list of all the projections with a description of the connectivity and the synapse model, a textual description of each neuron and synapse models used in the network (with the parsed equations) and finally the initial value of the parameters used in each population and projection. The generated file still requires some editing before being published, but it should ease the modeler's work.

## 3. Code generation

The approach chosen for the neural simulator is based on a complete code generation mechanism. As noted in Goodman (2010), code generation allows to couple the flexibility of a high-level language (here Python) with the speed and hardware specificities of a low-level language (C++). This approach is used in Brian to speed up some code portions and is further extended in Brian 2 where a complete C++code for the network can be optionally generated at runtime (cpp_standalone mode, Stimberg et al., 2014). ANNarchy relies entirely on this concept, by generating and compiling a shared C++ library during the call to compile(). Only this library will hold the data representing the model. The library is then imported by the Python script which transfers the initial value of all parameters and variables and starts the simulation. The Python script has only an indirect access to the C++ data and possible recordings through Cython wrappings. Cython is a Python-like compiled language allowing to execute instructions at C-speed and to access C or C++ data structures and methods (Behnel et al., 2009). Cython was for example used to create maintainable bindings to NEST (Zaytsev and Morrison, 2014).

The main advantage of a complete code generation in comparison to a simple interface to a low-level simulator (as in PyNest; Eppler et al., 2008) is that it allows to optimize the execution regarding the structure of the network. For example, if the model does not use delays in synaptic transmission (which require to implement queues for the output variables), or if no structural plasticity mechanism is involved (requiring more flexible data structures for the synapses), the corresponding code is not generated, reducing the complexity of the code and avoiding unnecessary overhead. Furthermore, the code can be adapted to the parallel computing platform, either a shared memory system with OpenMP (the parallel strategy can be different depending on whether 4 or 256 cores are available) or a graphical processing unit with CUDA (depending on its model or version). A drawback is that the structure of the network cannot be changed after the call to compile(): no population or projection can be added, or equations modified. The only changes possible are parameter or variable values, as well as the dynamical addition or suppression of synapses in case of structural plasticity.

### 3.1. Internal representation of data

Each population and projection is represented by a C++structure storing each attribute, either a parameter or a variable. Their name is easily extracted from the parameters and equations arguments to the neuron model: they are alone on the left side of the equation, except for ODEs where it is surrounded by d and /dt. Local attributes of a population are represented by a standard C++ vector with as many elements as neurons in the population while global ones (annotated by the population flag) are represented by a single value. Indexing is simple because all neurons have the same attributes.

For projections, the data representation depends on the platform: on shared memory systems with openMP, local attributes are represented by a vector of vectors, one per post-synaptic neuron receiving connections. Each of these vectors represents all synapses reaching this post-synaptic neuron (they can have different sizes). The connectivity matrix is therefore stored as a list of lists (LIL) structure in order to associate each value to the corresponding synapse. On graphical cards with CUDA, the connectivity is stored in the compressed sparse row (CSR) format, where the values of each attribute are flattened into a single vector and a list of row pointers allow to attribute portions of this array to a single post-synaptic neuron (see Brette and Goodman, 2011, for a review). These different data structures lead to a better parallel performance: CSR representations ensure a *coalesced* access to the attributes (i.e., the data is contiguous in memory), which is a strong condition for GPU computations to be efficient (Brette and Goodman, 2012), while the LIL structure allows a faster distribution of the data to the different OpenMP threads (Dinkelbach et al., 2012). LIL and CSR representations have similar memory requirements, but LIL is more adapted to the dynamical addition or suppression of synapses: structural plasticity is very inefficient on the GPU platform and is currently disabled.

The ability to adapt the data structures to the hardware is a clear advantage of the code generation approach, especially when the number and type of attributes is *a priori* unknown. These data structures can furthermore be easily exported to the Python namespace through the generation of Cython bindings, so the choice of the data structure is transparent to the user.

### 3.2. Simulation steps

ANNarchy performs the simulation with an equidistant time grid, where the integration step size dt is fixed for all equations. Although this scheme is natural for rate-coded networks, it can have a negative influence on spiking networks because of the forced alignment of spike times on this grid (Morrison et al., 2007). Brian also allows the use of different clocks for different parts of the model, which is currently impossible in ANNarchy. Future versions will address this issue.

Each simulation step is composed of several successive computational processes, which are mainly common to spiking and rate-coded networks:

1. *Propagation*: the results of the previous simulation step is propagated in the network. For rate-coded projections, the weighted sum of pre-synaptic firing rates is accumulated in the post-synaptic population. For spiking projections, the post-synaptic conductances are increased from the synaptic weight (or any other value defined in the pre_spike argument of the synapse) if the corresponding pre-synaptic neuron has emitted a spike. The variable updates defined in pre_spike are also processed if they exist (e.g., in the STDP rule). In both cases, if delays in synaptic transmission are defined, these operations are performed on the value of these variables at the corresponding time.
2. *Neural update*: the variables of each population are updated according to their definition in the equations argument of the neuron model. For spiking populations, the spiking condition is then evaluated. If the condition is met, the rank of the neuron is appended to a vector, the reset statement is evaluated and the neuron is possibly put into a refractory state. However, if a spiking neuron is in the refractory state, only the ODEs corresponding to the conductances are updated until the refractory period has elapsed, so no spike can be emitted.
3. *Delayed outputs*: before the simulation starts, each population computes the maximal delay in synaptic transmission required by outgoing projections and instantiates a double-ended queue of the adequate size. In this step, the new value of the output variable (firing rate or spike) is appended to the queue while the oldest value is removed.
4. *Synaptic updates*: the variables of each projection (if any) are updated, including synaptic plasticity.
5. *Post-synaptic events*: for each spiking projection where a post-synaptic neuron has emitted a spike, the post_spike statement is evaluated for all synapses reaching this neuron.
6. *Structural plasticity*: if structural plasticity is defined, the addition/suppression of synapses is evaluated.
7. *Recording*: each neural or synaptic variable is associated with a boolean flag which enables the recording of the variable with a given period. When the criterion is met, the value of the variable is appended to a vector.

Finally, the internal time t is incremented. These steps are all performed sequentially to ensure the correctness of the simulation. Parallel computations only occur within each of these steps if possible. The only difference between rate-coded and spiking networks are the pre_spike and post_spike statements, as well as the spike emission mechanism. This common structure allows hybrid networks to be simulated.

### 3.3. Mathematical parser

The different mechanisms described above are based on the equations defined at the neural or synaptic level. As the simulation is performed in C++, the computations are not vectorized, so an update rule for the variable has to be defined for each neuron of a population or each synapse of a projection. The transformation between the mathematical equation and the corresponding C++ code snippet is performed through the use of the Sympy library (Joyner et al., 2012) coupled with regular expressions.

The first step in the analysis of a neuron or synapse model is to determine with regular expressions the list of parameters and variables (by analysing the left side of the equation), their locality (presence of population or postsynaptic in the flags), their type (int, float or bool), bounds (min and max), initial value (init) and eventually the associated numerical method. The value of each parameter (e.g., tau = 10.0) is stored in a temporary dictionary which will be transferred to the C++library when it is instantiated.

For each variable, the equation is first manipulated to extract non-standard vocabulary. For example, the weighted sum in a rate-coded neuron (sum(exc)) is extracted and replaced by a temporary variable name (_sum_exc_). The same is done for random number distributions (Uniform(0, 1) is replaced by _rand_) and global operations (mean(pre.r) by _mean_pre_r). Conditional statements (if A: B else: C) are also extracted and each of the three terms are recursively analyzed. These temporary variables are added to the list of parameters and variables of the model.

This list allows to build a dictionary where the correspondence between the name of an attribute and its C++ equivalent is calculated. Each attribute belongs to a C++ structure representing a population or projection, so the name of the attribute must be prepended by the instance of the structure: pop%(id)s. for populations, proj%(id)s. for projections, where %(id)s will be replaced by the ID of the population or projection when the complete code is generated. As the update will be performed in a loop over all neurons or synapses, the index of the neuron in its population ([i]) or of the synapse in the projection ([i][j] for the LIL structure) is appended to this name. For example, the firing rate r of a neuron is represented by pop%(id)s.r[i] while the weight of a synapse becomes (proj%(id)s.w[i][j]).

Once the dictionary is built, Sympy is able to directly generate the C++ code equivalent to each side of the equation: constants (such as numbers) and functions of the C math library are automatically recognized and correctly translated. The temporary variables introduced for the weighted sums or random distributions are finally replaced by the adequate code thanks to regular expressions. As an example, the following equation for a neuron:


r = sum(exc) + B + cos(2*pi*t)


with B being a global parameter and t the current time in milliseconds, leads to the following code:


pop%(id)s.r[i] = pop%(id)s.sum_exc[i] + pop%(id)s.B
+ cos(2.0*M_PI*double(t)*dt))


### 3.4. Numerical methods

A special case has to be made for ODEs, as the desired numerical method will influence the resulting C++ code. Additionally, a neuron or synapse can be described by a set of coupled ODEs, so the code generation must be performed globally depending on the numerical method. We retained an approach similar to the one described in Stimberg et al. (2014), except that we do not explicitly generate an abstract code representation of the equations, but rather directly manipulate Sympy symbols.

To illustrate how the numerical methods are applied, we take the example of a simple spiking neuron defined by the Equation (13), but the principle is similar for synapses or rate-coded models, regardless of the number of ODEs.

(13) $$\begin{array}{l} \tau \cdot \frac{dv(t)}{dt}+v(t)=g_{\text{exc}}(t)-u(t)\\ \tau \cdot \frac{du(t)}{dt}+u(t)=v(t) \end{array}$$

**Figure 5:**
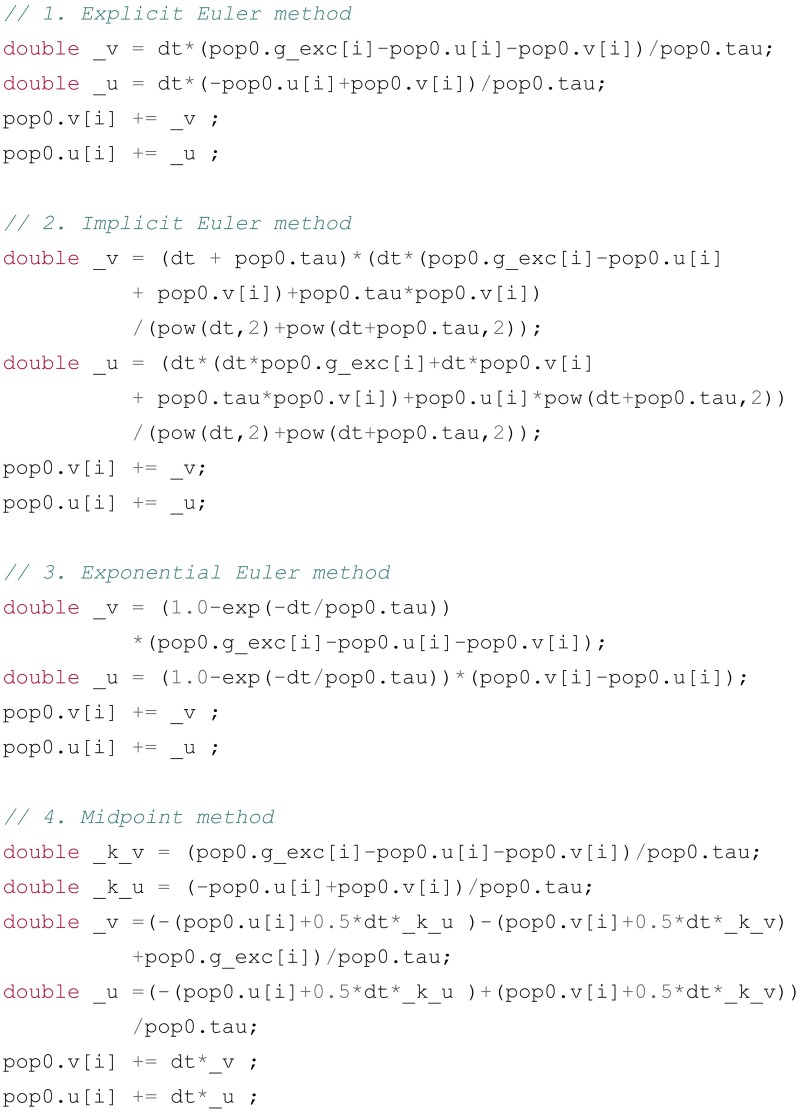
**Example of code generated for the Equation (13) using different numerical methods: 1. Explicit Euler; 2. Implicit Euler; 3. Exponential Euler; 4. Midpoint (Runge-Kutta method of order 2)**. pop0 is a C++ structure holding the different attributes of the population: the vectors v and u for the two variables, the vector g_exc for the excitatory inputs and the double value tau for the time constant. All methods compute first the increments _v and _u before adding them to v and u, in order to make sure the update rules use the previous values of these variables. The number of elementary operations differs from one method to another, increasing the simulation runtime, but the numerical precision and stability of themore complex methods might be required in some cases.

Such a neuron could be represented by the following description:


tau*dv/dt + v = g_exc - u
tau*du/dt + u = v


with tau being a global parameter of the population. The problem to be addressed by the numerical method is to find the next value of the variables v and u based on the value they had at the previous time step and the current value of the conductance g_exc. Figure 5 shows the code generated for these equations by the different available numerical methods (explicit, implicit, exponential and midpoint).

#### 3.4.1. Explicit euler

The explicit (or forward) Euler method evaluates the gradients dv/dt and du/dt at the current time *t*. In the textual representations of the equations, dv and du are simply replaced by two new variables _v and _u, and the system of equations is solved and simplified to find the value of these increments as a function of v, u, tau, and g_exc. Here, the problem is simple because _v and _u are present only once per equation: the equations are not coupled. The increments are translated into a C++code snippet using the same dictionary-based approach as for regular equations, and the increments are then added to the previous value of v and u.

#### 3.4.2. Implicit euler

The implicit (or backward) Euler method evaluates the gradients dv/dt and du/dt at the next time *t*+dt. dv and du are replaced by _v - v and _u - u, where _v and _u represent the next value of the variables, and all occurrences of v and u are replaced by _v and _u. This leads to a system of two linear equations with two variables, which is solved using the Sympy linear solver. Contrary to the explicit method, the equations are coupled, and the solver will only succeed if the equations are linear in v and u. The parser will return an error if not. Once the solution is found, we subtract v and u to _v and _u and simplify the equation in order to find the increment that will be added to the previous value of the variables.

#### 3.4.3. Exponential euler

The exponential Euler method is a special forward method which has the smallest numerical error on uncoupled linear first-order ODEs. The first step is to canonize each equation in the form $\tau \;\cdot \;\frac{dx(t)}{dt}+x(t)=A(t)$, with τ being the time constant of the variable and *A*(*t*) its steady state. Here the equations are already in this form, but a conductance-based neuron with the equation tau*dv/dt + v = g_exc*(E-v) would have an equivalent time constant of tau/(1+g_exc) and a steady state of g_exc*E/(1+g_exc). Once these equivalent time constants and steady states are identified and simplified for each equation, the increments can be directly obtained through:

(14) $$x(t+dt)=x(t)+(1-\exp(-\frac{dt}{\tau }))\cdot (A(t)-x(t)))$$

#### 3.4.4. Midpoint

The midpoint method is a Runge-Kutta method of order 2, described in Stimberg et al. (2014). It evaluates successively the gradient at *t* and in the middle of the interval [*t*, *t* + *dt*]. The gradient at *t* is evaluated using the same mechanism as in the explicit Euler method and stored in the variables _k_v and _k_u. These variables allow to estimate the value of v and u by v + dt/2*_k_v and u + dt/2*_k_u, respectively. The equations are again manipulated, by replacing all occurrences of v and u by their estimates at *t*+*dt*/2 and finding the corresponding increment using the explicit Euler method. This method has a much smaller numerical error and is more stable than the explicit or implicit methods, but requires more computations during the simulation, as the gradient is evaluated twice.

#### 3.4.5. Event-driven integration

This method is only available for spiking synapses, if the ODEs are linear (which is the case for the online STDP rule). For this method, the equations are not evaluated at each time step, but only when a pre- or post-synaptic spike occurs for a synapse. The new value of the variables is then computed exactly, using the time elapsed since the last event. Event-driven integration is not yet available for neural equations, as it requires to predict the occurrence of the next spike. Future versions of ANNarchy will address this mechanism. However, it may only speed simulations up if the network is small and does not generate too many spikes per step (Brette et al., 2007; Morrison et al., 2007).

### 3.5. OpenMP and CUDA code Generation

**Figure 6:**
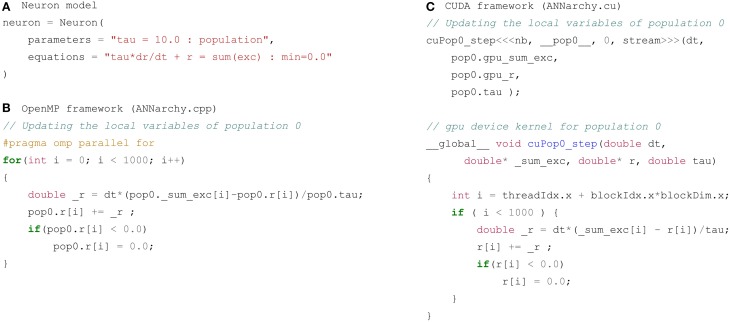
**Code generated for a single population pop0 of 1000 identical neurons**. **(A)** Neuron model used for code generation: a global parameter tau and a local variable r following a linear ODE and limited to positive values. **(B)** Code generated for the OpenMP framework. The code is pasted into the main C++ code ANNarchy.cpp and called at each step. It iterates over the 1000 neurons of the population and updates their firing rate depending on the corresponding code snippet. It operates directly on the data contained in the structure pop0. A simple #pragma statement allows parallel processing over the available threads. **(C)** Code generated for the CUDA framework. The code is pasted into the specific ANNarchy.cu file. A copy of the vectors _sum_exc and r (prefixed by gpu) is sent to the device (GPU) through the call to cuPop0_step by the host (CPU). The code inside cuPop0_step is executed in parallel on the device for the 1000 neurons and updates the array corresponding to r. This copy of r is transfered back to the CPU at the end of the simulation block for analysis in Python. Note that the parser can be configured to not generate the struct prefixes as for the OpenMP backend.

Once the structure of network is known and all equations have been analyzed, the C++code corresponding to the simulation can be generated depending on the desired parallel framework. Each simulation step described in Section 3.2 leads to the generation of a code portion for the corresponding populations and projections which is then integrated into the main simulation code. Figure 6 shows an example of a code portion for the update of the neural variables of a population pop0 whose 1000 neurons are defined by the neuron model described on Figure 6A. It defines a global parameter tau and the firing rate r is defined by the ODE tau*dr/dt = sum(exc) - r, limited to positive values with the flag min=0.0. The OpenMP implementation on Figure 6B is in this case simple: the code snippet corresponding to the ODE (here using the explicit Euler method) is integrated into a for-loop over the 1000 neurons, where the value of each element in the corresponding vector is updated sequentially. The parallel execution of this loop over the available cores is ensured through the addition of an OpenMP #pragma statement. The complete code is pasted in a standard C++file called ANNarchy.cpp and compiled using g++ on Linux or clang++ on MacOS X.

The code generated for the same population in the CUDA framework is more complex, as shown on Figure 6C. The instructions executed on the GPU have to be compiled with the NVIDIA compiler nvcc, so the code is generated in a special file called ANNarchy.cu. CUDA code generally consists of two sections: one is intended to run on the CPU (host code) while the other (flagged with the keywords __global__ or __device__) will be executed on the GPU (device code). At the beginning of the simulation, the vectors holding population and projection data are transferred to the GPU using the CUDA method cudaMemcpy(). The CUDA object will work on these copies during the whole simulation and they will be transfered back to the host at the end, allowing the Python script to analyze the results. An exception is during the recording of variables: the arrays to be recorded are transferred to the host at each time step, as the amount of memory is usually limited on GPUs.

Figure 6C shows the corresponding host and device code portions: the host code simply calls the device method with a copy of the necessary data. The device code updates the passed variables in parallel according to the desired numerical method. The same mechanism is used for all steps of the simulation. The weighted sum of inputs is for example executed in parallel over blocks of post-synaptic neurons with OpenMP. In contrast, parallel reduction is used in the CUDA implementation, as it leads to better performance (Dinkelbach et al., 2012). The main advantage of this code generation approach is that only the required steps are generated: spike-only mechanisms are skipped for rate-coded networks, as well as mechanisms for synaptic delays or structural plasticity if the network does not define them. This allows to minimize the code overhead and improves the readability of the generated code.

## 4. Benchmarks

We here report the parallel performance of the neural simulator but do not attempt to study it in all details. It is planned to issue future releases of ANNarchy, most improvements concerning the parallel performance. Nevertheless, we want to highlight that code generation already allows to obtain a parallel performance comparable to most specialized simulators. The OpenMP tests are performed on a Linux machine with 2 Intel XEON X5675 at 3 GHz (12 physical cores in total, with hyperthreading disabled) and 12 GB RAM. The CUDA tests are performed on a Linux machine with 2 Intel XEON E5-2650 at 2.6 GHz, 128 GB RAM and a NVIDIA Tesla K20m graphical card. The simulation times are measured and averaged over 10 different trials with the same initial conditions (standard deviations are omitted as they are negligible in all cases). All scripts used in this section are provided in the Supplementary Material.

### 4.1. Rate-coded benchmark

**Figure 7:**
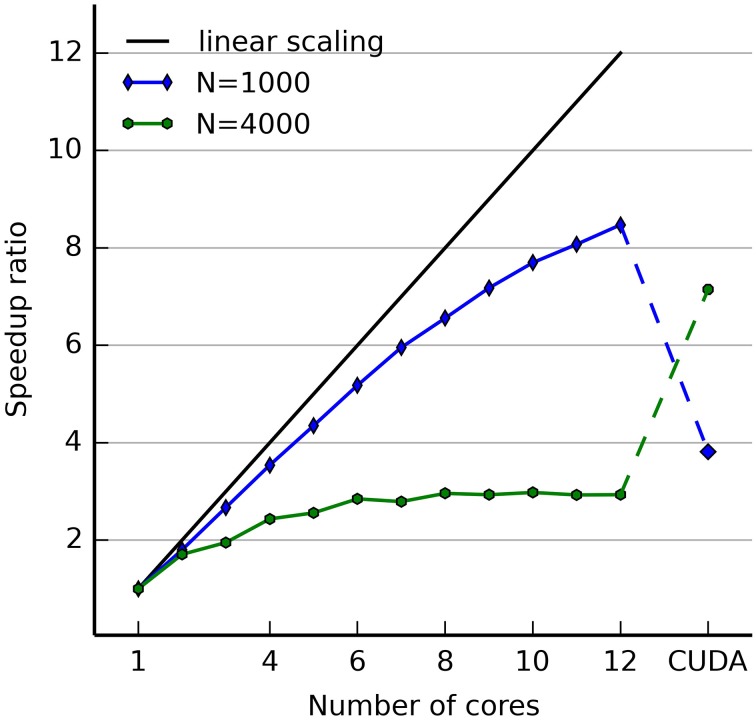
**Speedup ratio obtained by ANNarchy for a fully connected rate-coded network composed of two populations of 1000 (resp. 4000) neurons each**. The speedup ratio is defined by the ratio between the execution time (measured for a simulation of 1 s) of the single-threaded implementation and the one measured when using T threads. The single-threaded implementation does not use OpenMP nor CUDA primitives. For the OpenMP implementation, the number of threads is varied between 2 and 12. For the CUDA implementation, the default configuration of ANNarchy (32 threads for the neural variables updates, 192 threads for the weighted sums) is used. The CUDA implementation is run on a different machine for technical reasons, so the single-threaded baseline measured on this machine differs from the one used for OpenMP. Nevertheless, only the scaling ratio is interesting here, not the absolute execution times. The black line denotes the ideal linear scaling, the blue line the scaling of the network with 1000 neurons, the green one the scaling for 4000 neurons. With OpenMP, the scaling for 1000 neurons is slightly sub-optimal, while the one for 4000 neurons saturates quickly at a ratio of 2.9. The situation is reversed with CUDA: the network with 1000 neurons only achieves a speedup ratio of 3.8, while the network with 4000 neurons achieves a ratio of 7.15.

To test the parallel performance of rate-coded networks, we used a simple network of two populations composed of *N* = 1000 (resp. 4000) neuron each, connected with a all-to-all projection representing 1 (resp. 16) million connections. Each neuron is a simple leaky-integrator of excitatory inputs with a firing rate defined by the ODE tau*dr/dt + r = sum(exc), tau being a global parameter of the population. Unlike spiking networks, the simulation time of a rate-coded network does not depend on the activity in the network and the summation of inputs for all-to-all connectivity patterns hugely overcomes the update of neural variables (Dinkelbach et al., 2012), so such a simple network is sufficient to exhibit the parallel performance of the simulation. As outlined in the introduction, we are not aware of parallel simulators of rate-coded networks which could simply implement this network, so we only present in Figure 7 the speed-up ratio of the simulation time when using 1–12 threads with OpenMP or when using CUDA as the simulation backend. The single-threaded implementation is performed without the OpenMP primitives, so it avoids the small sequential overhead of OpenMP. The CUDA implementation uses the default configuration used by ANNarchy (32 threads for the neural variables updates, 192 threads for the weighted sums), but this can be changed by the user.

The network with 1000 neurons in each population shows a fairly efficient scaling behavior, while the network with 4000 neurons quickly saturates to a speed-up of approximately 2.9. This can be explained by the fact that the connectivity matrix with 16 million synapses (each connection weight being represented by a double floating-point value) cannot fit into the cache, so we have a memory-bound problem where memory transfers between the RAM and the processor limit the efficiency of the parallel implementation on shared-memory systems. This limitation is well-known for this kind of operation, especially because of the LIL structure used for the connectivity matrix. We chose this structure as it allows easier modification through structural plasticity mechanisms and internal tests showed that a CSR structure does not improve much the performance. We will investigate further the influence of data structures on parallel performance. The main operation performed here is a matrix-vector multiplication. The strategy to efficiently parallelize this operation depends on the sparseness of the connectivity matrix. Depending on this type, there are multiple methods available, including single-instruction-multiple-data operations (SIMD), cache blocking, loop unrolling, prefetching and autotuning (Williams et al., 2007; Kelefouras et al., 2015). Thanks to the code generation approach used in ANNarchy, we will be able in future versions to implement these improvements depending on the known connectivity before compilation.

The situation is reversed for the CUDA implementation: the network with 1000 neurons is speeded up by a factor 3.8, while the network with 4000 neurons obtains a speedup of 7.15, more than three times the maximal speedup obtained with OpenMP. This confirms our previous work showing that rate-coded networks with a relatively small number of connections might benefit more from a CPU-based implementation, while networks with many connections should be run on a GPU (Dinkelbach et al., 2012).

### 4.2. Spiking benchmark

For spiking networks, we compare the parallel performance of ANNarchy with other neural simulators on the COBA benchmark proposed in Brette et al. (2007) and based on the model of Vogels and Abbott (2005). The network is composed of 4000 integrate-and-fire neurons (3200 excitatory and 800 inhibitory) using exponentially-decreasing conductance-based synapses:

(15) $$\begin{array}{l} \;C\cdot \frac{dv(t)}{dt}=g_{L}\cdot (E_{l}-v(t))+g_{e}(t)\cdot (E_{e}-v(t))\\ \;+g_{i}(t)\cdot (E_{i}-v(t))+I\\ \tau_{e}\cdot \frac{dg_{e}(t)}{dt}=-g_{e}(t)\\ \tau_{i}\cdot \frac{dg_{i}(t)}{dt}=-g_{i}(t) \end{array}$$

All neurons are randomly connected with a probability of 0.02. We implemented this benchmark on ANNarchy (version 4.4.0), Brian (version 1.4.1), Brian 2 (version 2.0b3), NEST (with Python bindings, version 2.4.2), and Auryn (version 0.4.1). As noted in Zenke and Gerstner (2014), NEST uses by default the precise but very expensive Runge-Kutta-Fehlberg 4(5) (RK45) numerical method, while Brian and Auryn use the faster explicit Euler method. We therefore also applied the patch provided by Zenke and Gerstner (2014) to force NEST to use the Euler method (noted NEST-Euler as opposed to NEST-RK45). The Auryn simulator was modified to use synaptic delays of 0.1 ms. The code for Brian 2 uses the cpp_standalone mode to generate efficient C++ code and OpenMP parallel processing. All simulations were run using the same parameters, random number generator seeds (for the initial values of the membrane potential) and connectivity matrix (generated as a Scipy sparse matrix and loaded into the different simulators). The ANNarchy and Brian implementations produced exactly the same spiking patterns, while the other simulators showed only minor deviations. The time needed for 10 s of simulation (excluding building time) was measured using the Python time module, except for Auryn where MPI timer routines were used.

**Figure 8:**
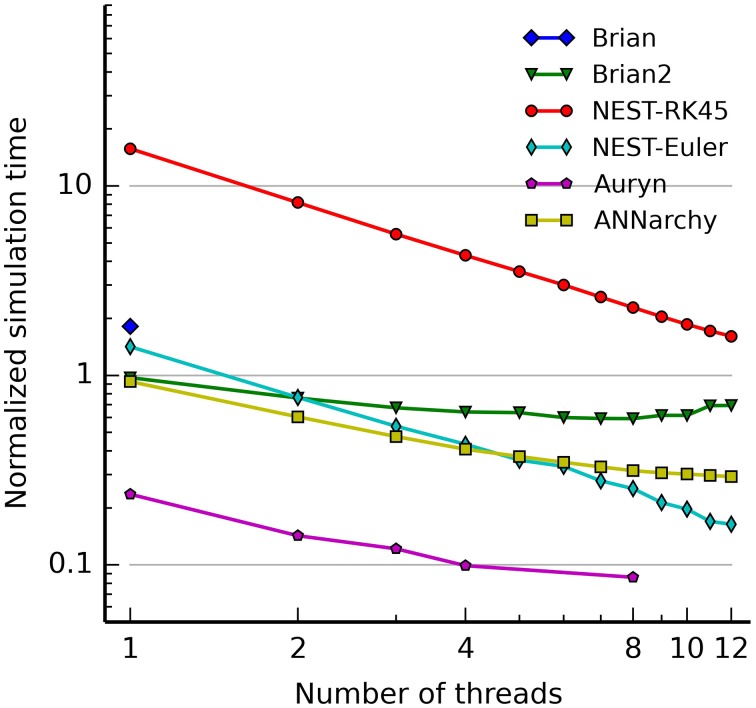
**Comparison of the simulation times of different simulators depending on the number of threads on a shared-memory system**. The parallel performance of the simulators Brian (version 1.4.1), Brian 2 (version 2.0b3), NEST (with Python bindings, version 2.4.2), Auryn (version 0.4.1), and ANNarchy (version 4.4.0) are investigated up to 12 threads. Two versions of NEST are used: one using the Runge-Kutta-Fehlberg 4(5) method (noted NEST-RK45), and a patched version using the explicit Euler method (NEST-Euler). The simulation times are normalized to show the real-time ratio: a normalized time of 1 means that simulating the network for 1 s takes exactly 1 s of computer time (simulations are run for 10 s). Both axes use a logarithmic scale. Brian only allows single-threaded simulations. Brian 2, NEST and ANNarchy use OpenMP, while Auryn uses MPI (openMPI 1.4.3). Auryn only allows a number of processes which is a multiple of 2. The single-threaded version of ANNarchy compares well to other neural simulators, but its scaling properties are not optimal compared to NEST.

The results are shown on Figure 8. In agreement with the results of Zenke and Gerstner (2014), the default NEST implementation with RK45 is roughly ten times slower than the modified NEST version with explicit Euler, but both have a very good scaling behavior. In the single-threaded version, Brian 2 is much faster than Brian and comparable to ANNarchy, but its scaling behavior is not as optimal as other simulators. It should be noted that Brian 2 is still in development, so this result is only preliminary. Auryn is almost one order of magnitude faster than the other simulators and with an satisfying scaling behavior (although the number of MPI processes must be a multiple of 2). The single-threaded implementation of ANNarchy is in comparison fairly efficient, but the scaling properties could be further improved. This is mostly due to the spike propagation mechanism (increasing post-synaptic conductances when a spike is emitted), which scales poorly in comparison to the neural variable updates. Future work will investigate different implementations of this mechanism.

## 5. Discussion

We have described the core principles of the neural simulator ANNarchy. It provides a high-level interface in Python similar to PyNN to facilitate the creation of rate-coded, spike-coded or hybrid neural networks. An important set of neuron and synapse models can be implemented with an equation-oriented syntax close to the one proposed by Brian. These definitions are used to generate an entire C++library optimized for the underlying parallel framework (OpenMP for shared memory systems, CUDA for GPU cards). Different numerical methods are available for solving the possible ODEs. Code generation allows complete control over data structures and computational methods, which leads to the execution of fine-tuned and simple code. It allows to obtain a parallel performance comparable to specialized simulators.

ANNarchy brings the flexibility of the Brian interface to rate-coded networks, while being compatible with state-of-the-art spiking simulators. Although several features and concepts for spiking networks are comparable to other simulators (especially Brian 2, Stimberg et al., 2014), ANNarchy also provides novel features to the community. Structural plasticity can be easily implemented through simple synapse-specific rules. Any neural or synaptic variable can be easily recorded during the simulation. The network can be easily interfaced to external C/C++libraries through the Cython bindings, so images or video streams can efficiently be fed to the network, or neural activity read to control robots in real-time. Automatic reporting allows to generate complete reports in 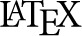 about the current network model, including the network structure, the equations used for the neurons and synapses, as well as the different parameters used. Brian 2 provides a similar feature as it is also based on Sympy, but only for individual equations. Some features are implemented only for rate-coded networks (such as convolution or pooling operations which do not make much sense for spiking networks), but the hybrid ability of ANNarchy allows for example to integrate convoluted rate-coded networks for vision with spiking cognitive models.

The chosen equation-oriented approach is very powerful, but has some limitations, some of which are already listed in Stimberg et al. (2014). The number of explicit neural states is limited to two for spiking neurons (active or refractory) and only one for rate-coded ones. However, the syntax allows the use of conditional statements which can modify entirely the properties of a neuron, mimicking additional states. The equation-oriented syntax is also limited in its current form to the description of point-neurons, neglecting the effects of the neurons' morphology on their properties. Such neurons would require the use of another simulator such as NEURON or GENESIS.

As Brian 2 and ANNarchy are based on the principles stated in Stimberg et al. (2014), one should highlight the main differences between the two equation-oriented interfaces for spiking networks. Brian 2 proposes a powerful mechanism to incrementally build connection matrices by accessing the underlying data structure, possibly through text-based rules. It is also possible to dynamically add and remove populations and projections between two simulations. This is currently impossible with ANNarchy: all data structures are linked to the generated library and are only indirectly accessible in Python. Synapse definition in Brian 2 allows to modify any pre- or post-synaptic neural variable. Because of the way the code is generated, ANNarchy only allows the synapse to modify the post-synaptic conductance in addition to synaptic variables. Brian 2 allows to solve stochastic differential equations (SDE), while ANNarchy is limited for now to ODEs: one can only use random variables inside an ODE to simulate for example intracellular noise, but this is not a stochastic process. Brian 2 allows a finer control on the evolution of neural variables during the refractory period, while ANNarchy freezes all variables during this period except for the conductances. SDEs and control over variables during the refractory period will be progressively introduced in future versions. On the other hand, ANNarchy proposes a solution to structural plasticity and hetero-synaptic plasticity (through the possible use of global post-synaptic variables in a projection) which could be integrated in Brian 2. It also provides additional control over the evolution of variables, such as their initial value and the minimal or maximal value they can take over the course of a simulation.

ANNarchy will be further maintained and new features will be integrated in future releases. Learning in rate-coded networks is focused on biologically-plausible rules where all information is local to the synapse, which currently rules out methods such as backpropagation. Synaptic delays are currently only implemented between the pre-synaptic neuron and the synapse, while some plasticity models rely on an additional delay between the synapse and the soma of the post-synaptic neuron. Exact event-based integration of neural dynamics needs to be implemented (Morrison et al., 2007), as it allows to simulate faster low-firing networks of linear neurons. Additional numerical methods (such as Runge-Kutta of order 4) will be progressively introduced. Computations are limited to an equidistant time grid, as it is the easiest method for rate-coded networks. Some networks may nevertheless benefit from adaptive time steps, or of the use of different clocks in different parts of the model. This may be particularly useful for hybrid networks, as rate-coded networks often behave well with integration steps of 1 ms, while some spiking networks require at least 0.1 ms. Finally, as the chosen interface is very close to PyNN (Davison et al., 2008), we will implement a fully compatible interface so that ANNarchy can be used as an alternative simulation backend using the available standard models.

As the interface is already stable, there is room for improvement regarding the parallel performance. On CPU-based shared memory systems, the OpenMP implementation is efficient for rate-coded networks (in the limit of memory bandwidth), but the spike propagation mechanism does not scale linearly yet, introducing a strong sequential component to the simulation. This issue will be investigated in future releases: based on our experiments, simulators using array-based computations (Brian 2, ANNarchy and partially Auryn) tend to scale sub-optimally, while NEST performs better. A possible reason for this difference is linked to the object-oriented design of NEST: each thread computes individual neurons, leading to a more cache-friendly access to the variables, especially when using synaptic delays. In contrast the array-based approach share neural and synaptic data among several threads and quickly fill the cache. The opposite effect seems to be true for the update of neural variables (Zenke and Gerstner, 2014). Hybrid solutions between array-based and object-oriented implementations might lead to a better parallel performance for spiking networks.

Parallel computing on distributed memory systems is also planned. The performance of NEST on such systems suggests that this is an interesting solution for spiking networks, although it has been shown that memory transfers might impair scaling already for medium-scale spiking networks (Zenke and Gerstner, 2014). Communication costs might become a problem for rate-coded networks, as firing rates must be exchanged at each simulation step. However, if synaptic data is appropriately distributed on each node, it may increase the total available memory bandwidth, which is an important limiting factor. We are currently investigating hybrid MPI/OpenMP solutions which may minimize the communication costs through a structural analysis of the network's topology.

The generation of CUDA code for simulation on GPU platforms is still experimental and currently only available for rate-coded networks. One major issue is the choice of the correct configuration depending on the network, such as the number of threads per operation (the optimal number of threads for the summation of inputs is different from the one for the update of neural or synaptic variables). ANNarchy currently proposes a default configuration which can be overwritten by the user, but we will investigate solutions using auto-tuning of the simulation parameters (Dinkelbach et al., 2012).

## Author contributions

JV and HD designed and wrote the library. JV wrote primarily the article and performed the tests. FH supervised the development and participated in the writing.

### Conflict of interest statement

The authors declare that the research was conducted in the absence of any commercial or financial relationships that could be construed as a potential conflict of interest.

## Footnotes

^1^The GeNN project, http://sourceforge.net/projects/genn.

^2^http://bitbucket.org/annarchy/annarchy and http://annarchy.readthedocs.org.
