# Supplementary material for: ANNarchy: a code generation approach to neural simulations on parallel hardware
Source: Front Neuroinform. 2015 Jul 31;9:19. doi: 10.3389/fninf.2015.00019 (PMC4521356; doi:10.3389/fninf.2015.00019)
Supplement: Supplementary file 1 [file DataSheet1.PDF]

Frontiers in Neuroinformatics

Research Article
25 June 2015

Supplementary Material: ANNarchy: a code generation approach to neural simulations on parallel hardware

Julien Vitay [1,*], Helge Ülo Dinkelbach [1] and Fred H. Hamker [1,2]

[1] *Department of Computer Science, Chemnitz University of Technology, Chemnitz, Germany*
[2] *Bernstein Center for Computational Neuroscience, Charité University Medicine, Berlin, Germany*

Correspondence*:
Julien Vitay
Fakultät für Informatik, Professur Künstliche Intelligenz, Technische Universität Chemnitz, Straße der Nationen 62, D-09107 Chemnitz, Germany,
julien.vitay@informatik.tu-chemnitz.de

1 HYBRID NETWORKS

The following script allows to reproduce Fig. 4 of the manuscript.

```
from ANNarchy import *
setup(dt=0.1)
# Rate-coded input neuron
input_neuron = Neuron(
    parameters = "baseline = 0.0",
    equations = "r = baseline"
)
# Rate-coded output neuron
simple_neuron = Neuron(
    equations = "r = sum(exc)"
)
# Rate-coded population for input
pop1 = Population(geometry=1, neuron=input_neuron)
# Poisson Population to encode
pop2 = PoissonPopulation(geometry=1000, target="exc")
proj = Projection(pop1, pop2, "exc").connect_all_to_all(weights=1.)
# Rate-coded population to decode
pop3 = Population(geometry=1000, neuron =simple_neuron)
proj = DecodingProjection(pop2, pop3, "exc", window=10.0)
def connector(pre, post, weights):
    csr = CSR()
    for rk_post in range(post.size):
        csr.add(rk_post, range((rk_post+1)), [weights], [0] )
    return csr
proj.connect_with_func(method=connector, weights=1.)
compile()
# Monitors
m1 = Monitor(pop1, "r")
```

```
m2 = Monitor(pop2, "spike")
m3 = Monitor(pop3, "r")
# Simulate
duration = 250.
# 0 Hz
pop1.baseline = 0.0
simulate(duration)
# 10 Hz
pop1.baseline = 10.0
simulate(duration)
# 50 Hz
pop1.baseline = 50.0
simulate(duration)
# 100 Hz
pop1.baseline = 100.0
simulate(duration)
# Get recordings
data1 = m1.get()
data2 = m2.get()
data3 = m3.get()
# Raster plot of the spiking population
t, n = m2.raster_plot(data2["spike"])
# Variance of the the decoded firing rate
data_10 = data3["r"][1.0*duration/dt():2*duration/dt(), :]
data_50 = data3["r"][2.0*duration/dt():3*duration/dt(), :]
data_100 = data3["r"][3.0*duration/dt():4*duration/dt(), :]
var_10 = np.mean(np.abs((data_10 - 10.)/10.), axis=0)
var_50 = np.mean(np.abs((data_50 - 50.)/50.), axis=0)
var_100 = np.mean(np.abs((data_100 - 100.)/100.), axis=0)
### Plot the results
from pylab import *
subplot(3,1,1)
plot(t, n, ".", markersize=0.5)
title("a) Raster plot")
xlabel("Time (ms)")
ylabel("# neurons")
xlim((0, 4*duration))
subplot(3,1,2)
plot(np.arange(0, 4*duration, 0.1), data1["r"][:, 0], label="Original firing rate")
plot(np.arange(0, 4*duration, 0.1), data3["r"][:, 999], label="Decoded firing rate")
legend(frameon=False, loc=2)
title("b) Decoded firing rate")
xlabel("Time (ms)")
ylabel("Activity (Hz)")
subplot(3,1,3)
plot(var_10, label="10 Hz")
plot(var_50, label="50 Hz")
plot(var_100, label="100 Hz")
legend(frameon=False)
title("c) Precision")
xlabel("# neurons used for decoding")
ylabel("Normalized error")
ylim((0,1))
show()
```

2 PERFORMANCE TESTS FOR RATE-CODED NETWORKS

The following script allows to simulate the rate-coded network used for the performance test depicted on Fig. 7 of the manuscript. The number of threads is controlled by passing the `-jX` argument to the Python call, where X is the number of threads.

```
from ANNarchy import *
import time
# Uncomment to use CUDA
#setup(paradigm="cuda")
# Default rate-coded neuron
LeakyNeuron = Neuron(
    parameters="tau = 10.0 : population",
    equations="tau*dr/dt + r = sum(exc) : min=0.0"
)
# Number of neurons
N = 1000 # 4000
# Creating the populations
Input  = Population(geometry=N, neuron=LeakyNeuron)
Output = Population(geometry=N, neuron=LeakyNeuron)
# Creating the projection
proj = Projection(Input, Output, "exc")
proj.connect_all_to_all(weights = Uniform(0.0, 1.0) )
# Compile the network
compile()
# Measure simulation time
t_start = time.time()
simulate(1000.0)
duration = time.time() - t_start
print "Done in", duration
```

3 PERFORMANCE TESTS FOR SPIKING NETWORKS

We provide here the ANNarchy, Brian, Brian2, NEST and Auryn scripts allowing to reproduce the Fig. 8 of the manuscript. All scripts use the same seed for the random number generators, and the connectivity matrix is loaded from two files `"exc.data"` and `"inh.data"` for the excitatory and inhibitory connections. Monitoring/recording was disabled for all simulators. The connectivity matrices were generated by creating the corresponding Scipy sparse matrices (in LiL format) and saved to disk using cPickle. Auryn requires the connectivity matrices to be a compressed-sparse row (CSR) matrix saved in the MatrixMarket format. The following code allows to generates these files:

```
from scipy.sparse import lil_matrix, csr_matrix
from scipy.io import mmwrite
from random import sample
import numpy as np
import cPickle
# Parameters
NE = 3200
NI = 800
sparseness = 0.02
# For ANNarchy, Brian and NEST
We=lil_matrix((NE, NE+NI))
Wi=lil_matrix((NI, NE+NI))
for i in xrange(NE):
    k = np.random.binomial(NE+NI, sparseness, 1)[0]
```

```
    We.rows[i] = sample(xrange(NE+NI), k)
    We.data[i] = [1.0]*k
for i in xrange(NI):
    k = np.random.binomial(NE+NI, sparseness, 1)[0]
    Wi.rows[i] = sample(xrange(NE+NI), k)
    Wi.data[i] = [1.0]*k
with open("exc.data", "w") as wfile:
    cPickle.dump(We, wfile)
with open("inh.data", "w") as wfile:
    cPickle.dump(Wi, wfile)
# For Auryn
we = 0.6
wi = 6.7
We_full = lil_matrix((NE+NI, NE+NI))
Wi_full = lil_matrix((NE+NI, NE+NI))
for i in xrange(NE):
    We_full.rows[i] = We.rows[i]
    We_full.data[i] = We.data[i]
for i in xrange(NI):
    Wi_full.rows[i+NE] = Wi.rows[i]
    Wi_full.data[i+NE] = Wi.data[i]
We_full = csr_matrix(we*We_full)
Wi_full = csr_matrix(wi*Wi_full)
mmwrite("exc.wmat", We_full)
mmwrite("inh.wmat", Wi_full)
```

3.1 ANNARCHY

The ANNarchy script for the COBA benchmark is the following:

```
from ANNarchy import *
# Configuration
import time, cPickle
setup(dt=0.1, seed=98765)
# COBA neuron
COBA = Neuron(
    parameters="""
        El = -60.0          : population
        Vr = -60.0          : population
        Erev_exc = 0.0      : population
        Erev_inh = -80.0    : population
        Vt = -50.0          : population
        tau = 20.0          : population
        tau_exc = 5.0       : population
        tau_inh = 10.0      : population
        I = 20.0            : population
    """,
    equations="""
        tau*dv/dt = (El - v) + g_exc*(Erev_exc - v) + g_inh*(Erev_inh - v) + I
        tau_exc * dg_exc/dt = - g_exc
        tau_inh * dg_inh/dt = - g_inh
    """,
    spike = "v > Vt",
    reset = "v = Vr",
    refractory = 5.0
)
# Populations
```

```
P = Population(geometry=4000, neuron=COBA)
Pe = P[:3200]
Pi = P[3200:]
P.v = Normal(-55.0, 5.0)
# Connections
we = 0.6
wi = 6.7
mate = cPickle.load(open("exc.data", "r"))
mati = cPickle.load(open("inh.data", "r"))
Ce = Projection(pre=Pe, post=P, target="exc")
# Ce.connect_fixed_probability(weights=we, probability=0.02)
Ce.connect_from_sparse(weights=mate*we)
Ci = Projection(pre=Pi, post=P, target="inh")
# Ci.connect_fixed_probability(weights=wi, probability=0.02)
Ci.connect_from_sparse(weights=mati*wi)
# Compile and simulate
compile()
start_time = time.time()
simulate(10000.0)
duration = time.time() - start_time
```

3.2 BRIAN

The brian script is directly adapted from their example http://www.briansimulator.org/docs/examples-misc_COBA.html.

```
from brian import *
# Configuration
import numpy, time, cPickle
numpy.random.seed(98765)
set_global_preferences(useweave=True)
set_global_preferences(usecodegen=True)
# Parameters
taum = 20 * msecond
taue = 5 * msecond
taui = 10 * msecond
Ee = 0. * mvolt
Ei = -80. * mvolt
El = - 60.0 * mvolt
I = 20. * mvolt
# Equations
eqs = Equations("""
dv/dt = (El-v + ge*(Ee-v) + gi*(Ei-v) + I) * (1./taum) : volt
dge/dt = -ge*(1./taue) : 1
dgi/dt = -gi*(1./taui) : 1
""")
# Populations
P = NeuronGroup(4000, model=eqs, threshold=-50 * mvolt, \
              reset=-60 * mvolt, refractory=5 * msecond,
              order=1, compile=True)
Pe = P.subgroup(3200)
Pi = P.subgroup(800)
P.v = (randn(len(P)) * 5. - 55.) * mvolt
# Connections
we = 0.6
wi = 6.7
mate = cPickle.load(open("exc.data", "r"))
```

```
mati = cPickle.load(open("inh.data", "r"))
Ce = Connection(Pe, P, "ge")
# Ce.connect_random(weight=we, p=0.02)
Ce.connect_from_sparse(mate*we, column_access=False)
Ci = Connection(Pi, P, "gi")
# Ci.connect_random(weight=wi, p=0.02)
Ci.connect_from_sparse(mati*wi, column_access=False)
# Simulate
start_time = time.time()
run(10 * second)
duration = time.time() - start_time
```

3.3 BRIAN2

The Brian2 script is directly adapted from their example `http://brian2.readthedocs.org/en/2.0beta/examples/standalone.cuba_openmp.html`. It uses the `cpp_standalone` mode allowing complete code generation and parallel processing using OpenMP. The number of OpenMP threads is controlled by setting the `prefs.devices.cpp_standalone.openmp_threads` variable.

Run-time measurements are done by comparing the measured times in two separate scripts: one running the network for 0 second, the other for 10 seconds. This is because the `cpp_standalone` mode generates and eventually compiles the generated code for each call to `device.build()` (as the `compile()` method of ANNarchy does), and transfer the connectivity matrices to the C++ executable. The difference between the two durations represents the real time needed for the 10 seconds simulation.

```
from brian2 import *
# Configuration
import numpy, time, cPickle
numpy.random.seed(98765)
set_device("cpp_standalone")
prefs.devices.cpp_standalone.openmp_threads = 1
# Parameters
taum = 20 * ms
taue = 5 * ms
taui = 10 * ms
Vt = -50 * mV
Vr = -60 * mV
El = -60 * mV
Erev_exc = 0 * mV
Erev_inh = -80 * mV
I = 20 * mvolt
# Equations
eqs = """
dv/dt = (ge*(Erev_exc-v)+gi*(Erev_inh-v)-(v-El)+I)*(1./taum) : volt (unless refractory)
dge/dt = -ge/taue : 1
dgi/dt = -gi/taui : 1
"""
# Populations
P = NeuronGroup(4000, eqs, threshold="v>Vt", reset="v = Vr",
                refractory=5*ms, method="euler")
P.v = (randn(len(P)) * 5. - 55.) * mvolt
# Connections
we = 0.6
wi = 6.7
Ce = Synapses(P, P, pre="ge += we")
```

```
Ci = Synapses(P, P, pre="gi += wi")
#Ce.connect("i<3200", p=0.02)
#Ci.connect("i>=3200", p=0.02)
Ce_pre, Ce_post = cPickle.load(open("exc.data", "r")).nonzero()
Ci_pre, Ci_post = cPickle.load(open("inh.data", "r")).nonzero()
Ce.connect(Ce_pre, Ce_post)
Ci.connect(Ci_pre+3200, Ci_post)
# Simulate
t1 = time.time()
run(10. * second)
device.build(directory="COBA", compile=True, run=True, debug=False)
t2 = time.time()
duration = t2 - t1
```

3.4 NEST

The NEST script for Python is adapted from the one used in Zenke and Gerstner (2014). The number of OpenMP threads is controlled through the `local_num_threads` variable.

```
from nest import *
# Configuration
import numpy, time, cPickle
numpy.random.seed(98765)
SetKernelStatus({"resolution": 0.1})
SetKernelStatus({"local_num_threads": 1})
# Parameters
NE      =  3200
NI      =   800
# Populations
SetDefaults("iaf_cond_exp", {
    "C_m"  : 200.,
    "g_L": 10.,
    "tau_syn_ex": 5.,
    "tau_syn_in": 10.,
    "E_ex": 0.,
    "E_in": -80.,
    "t_ref": 5.,
    "E_L"  : -60.,
    "V_th" : -50.,
    "I_e": 200.,
    "V_reset"   : -60.,
    "V_m"       : -60.
})
nodes_ex = Create("iaf_cond_exp", NE)
nodes_in = Create("iaf_cond_exp", NI)
nodes = nodes_ex+nodes_in
v = -55.0 + 5.0*numpy.random.normal(size=NE+NI)
for i, node in enumerate(nodes):
    SetStatus([node], {"V_m": v[i]})
# Projections
w_exc = 6.
w_inh = -67.
SetDefaults("static_synapse", {"delay": 0.1})
CopyModel("static_synapse", "excitatory", {"weight": w_exc})
CopyModel("static_synapse", "inhibitory", {"weight": w_inh})
mate = cPickle.load(open("exc.data", "r"))
mati = cPickle.load(open("inh.data", "r"))
```

```
# Connect(nodes_ex, nodes,{"rule": "pairwise_bernoulli", "p": 0.02}, syn_spec="excitatory")
for i in range(NE):
    post = list(mate.rows[i])
    Connect([nodes_ex[i]], [nodes[p] for p in post], "all_to_all", syn_spec="excitatory")
# Connect(nodes_in, nodes,{"rule": "pairwise_bernoulli", "p": 0.02}, syn_spec="inhibitory")
for i in range(NI):
    post = list(mati.rows[i])
    Connect([nodes_in[i]], [nodes[p] for p in post], "all_to_all", syn_spec="inhibitory")
# Simulate
start_time = time.time()
Simulate(10000.0)
duration = time.time() - start_time
```

4 AURYN

Auryn uses a C++ file to describe the network, which is then compiled with the source code of the simulator. We simplified the example provided in the source code by removing all monitoring and sanity checks. The parameter values for the network are already correctly defined in the source code of `TIFGroup`. We did not find a way to initialize the membrane potential with the same seed as for the other simulators, but it does not influence the simulation times significantly. The number of processes is controlled by using the OpenMPI `mpirun` executable. Measuring the simulation times required to put a synchronization barrier before and after the simulation call, while measuring the elapsed time on the first process only.

```
#include "auryn.h"
#include "mpi.h"
using namespace std;
namespace mpi = boost::mpi;

int main(int ac, char *av[]) {
    mpi::environment env(ac, av);
    mpi::communicator world;
    communicator = &world;
    logger = new Logger("/tmp/coba.log", world.rank(), PROGRESS, EVERYTHING);
    sys = new System(&world);
    // Population
    TIFGroup * neurons = new TIFGroup(4000);
    neurons->set_state("bg_current", 2e-2);
    neurons->random_mem(-55e-3,5e-3);
    // Connections
    SparseConnection * con_e = new SparseConnection(neurons, neurons, "exc.wmat", GLUT);
    SparseConnection * con_i = new SparseConnection(neurons, neurons, "inh.wmat", GABA);
    // Simulate
    int rank = -1;
    MPI_Comm_rank(MPI_COMM_WORLD, &rank);
    double start_time, duration;
    if (rank == 0) start_time = MPI_Wtime();
    MPI_Barrier(MPI_COMM_WORLD);
    bool result = sys->run(10.0, false);
    MPI_Barrier(MPI_COMM_WORLD);
    if (rank == 0) duration = MPI_Wtime() - start_time;
    // Quit
    delete sys;
    return 0;
}
```
